# Assessment of Spontaneous Neuronal Activity *In Vitro* Using Multi-Well Multi-Electrode Arrays: Implications for Assay Development

**DOI:** 10.1523/ENEURO.0080-19.2019

**Published:** 2020-01-22

**Authors:** Joseph Negri, Vilas Menon, Tracy L. Young-Pearse

**Affiliations:** 1 Ann Romney Center for Neurologic Diseases, Brigham and Women’s Hospital, Harvard Medical School, Boston, MA 02115; 2 Graduate Program in Biological and Biomedical Sciences, Division of Medical Sciences, Harvard University, Cambridge, MA 02138; 3 Department of Neurology, Columbia University Medical Center, New York, NY 10032

**Keywords:** multi-electrode arrays, spike sorting

## Abstract

Multi-electrode arrays (MEAs) are being more widely used by researchers as an instrument platform for monitoring prolonged, non-destructive recordings of spontaneously firing neurons *in vitro* for applications in modeling Alzheimer’s, Parkinson’s, schizophrenia, and many other diseases of the human CNS. With the more widespread use of these instruments, there is a need to examine the prior art of studies utilizing MEAs and delineate best practices for data acquisition and analysis to avoid errors in interpretation of the resultant data. Using a dataset of recordings from primary rat (*Rattus norvegicus*) cortical cultures, methods and statistical power for discerning changes in neuronal activity on the array level are examined. Further, a method for unsupervised spike sorting is implemented, allowing for the resolution of action potential incidents down to the single neuron level. Following implementation of spike sorting, the dynamics of firing frequency across populations of individual neurons and networks are examined longitudinally. Finally, the ability to detect a frequency independent phenotype, the change in action potential amplitude, is demonstrated through the use of pore-forming neurotoxin treatments. Taken together, this study provides guidance and tools for users wishing to incorporate multi-well MEA usage into their studies.

## Significance Statement

Multi-electrode arrays (MEA) are an instrument platform being used by an increasing number of neuroscientists for the purpose of monitoring spontaneous firing of neurons *in vitro* over extended periods of time. Through an analysis of existing literature and empirically generated datasets, this study seeks to establish best practices for the use of these instruments for applications employing neuronal cultures. Elements of experimental design and analysis for assaying firing frequencies across arrays are discussed, with a focus on the use of multi-well MEAs. Additionally, methods for (1) resolving signal to individual neurons through unsupervised spike-sorting, (2) assessing network dynamics, and (3) quantifying changing in action potential amplitudes are reported.

## Introduction

Among the conditions posing the greatest unmet medical need are diseases of the CNS. Confounding efforts toward the development of therapies, these conditions occur within an organ system that is largely inaccessible to direct experimentation and thus studies must be performed using experimental models. Attempting to study any disease or biological process in model systems always comes with inherent compromises. The system must be sufficiently complex to mimic the multiple facets of the *in vivo* physiology. Yet the system must be sufficiently reductionist to: (1) have clearly defined end-points, (2) be executable within reasonable timeframes, and (3) allow for parallel testing of multiple perturbations. Attempts to model these complex systems has motivated the field of therapeutic discovery to increasingly adopt representative phenotypic assays that: (1) use the most relevant cell type, (2) employ a disease relevant perturbation, (3) and rely on an assay metric that is representative of the disease symptomology (Vincent et al., 2015).

An assay platform for the study of the CNS and associated disorders must fulfill several criteria. It must be capable of capturing neuronal activity and do so within dense, mixed cultures of neurons and glia. The method should generate a rich dataset to allow for the detection of modest changes in phenotypes, particularly when perturbed at physiologically relevant magnitudes which may result in only small yet meaningful responses. Moreover, the method should be amenable to incorporate human cellular models, drawing on advancements in differentiation efficiency of human induced pluripotent stem cell (iPS) derived neurons and glia. While numerous assay platforms are employed in the field of neuroscience for assaying neuronal behavior, one which is capable of fulfilling all of these criteria are multi-electrode arrays (MEAs).

MEAs are instruments that provide a means of monitoring spontaneous electrophysiological activity within *in vitro* neuronal cultures. MEA consist of a large number (dozens to hundreds) of planar electrodes embedded in the base of a tissue culture chamber that allow for the parallel detection of local field potentials generated by the spontaneous or evoked firing of neurons. MEAs sample the potential difference across recording and reference electrodes at high rates (10–60 kHz), and action potentials (spikes) are detected when the sampled values deviate substantially from the background potential. Activity within cultures is quantified by the frequency of spiking events, and in some applications the occurrence of synchronized spiking events, or “bursts” (Spira and Hai, 2013; Obien et al., 2014).

By monitoring endogenously generated voltage potentials of firing neurons, these instruments do not rely on fluorescent dyes or proteins and microscopy apparatus required of calcium-imaging techniques. Additionally, MEAs are able to record simultaneously across upwards of hundreds of channels in a much less labor-intensive manner than conventional single-channel electrophysiological techniques such as patch-clamping or sharp-electrode recording, albeit with a lower degree of spatial resolution. As MEA recordings are non-destructive to the cultures, repeated recordings may be performed for as long as culture integrity can be maintained. For these reasons, MEA use is becoming more common in neuroscience and biomedical research.

Despite the long history and increased use of MEAs, methodologies are still being developed across the field. To date, the most comprehensive methodological review of *in vitro* MEA techniques was performed by Novellino et al. (2011). Published in 2011, that study assessed the use of single-well MEAs, single chamber vessels typically with ∼60 recording electrodes. Over the last decade, several multi-well MEAs systems have become commercially available, including those from Axion Biosystems, Alpha Med Scientific, Multichannel Systems, and MaxWell Biosystems. These multi-well MEAs adhere to the form factors of tissue culture plates, with a separate array occupying each well (we will subsequently equate a single well of electrodes as an array). The availability of multi-well MEAs has allowed for changes in experimental design and analysis, with the capability to test more conditions in parallel and use array-level (as opposed to electrode-level) activity metrics.

Accounting for these developments, this study sought to extend best practices for multi-well MEA experiments. Following a review of the literature, commonly reported experimental conditions and spike frequency analysis methods where evaluated through an empirically generated dataset. These data were leveraged to develop a method of cohort assignment for multi-well MEA experiments that accounts for inherent variability in firing frequencies. Further, implementation of unsupervised spike sorting to resolve activity to the level individual neurons (single-units) demonstrates how MEA recording data may be extended for examination of wave form amplitude and functional network phenotypes.

## Materials and Methods

### Tissue culture

#### Preparation of MEA plates

We prepared 96-well multi-electrode plates (Axion Biosystems) before the addition of cells by coating with poly-Ornithine (poly-O), laminin (both Sigma-Aldrich), and Matrigel (Corning Life Sciences). Briefly, 1 d before establishment of cultures, a solution of 20 μg/ml poly-O: 5 μg/ml laminin in 1× PBS was added to the MEA plates in a volume of 60 μl per well, and incubated overnight at 37°C. The day of culture plating, the poly-O:laminin solution was aspirated and the plates were washed once with 1× PBS. Matrigel was reconstituted 1:20 in ice-cold DMEM culture media (Gibco; ThermoFisher) without added serum or antibiotics and passed through a 40-μm strainer (BD Falcon). The Matrigel solution was added to the MEA plates in a volume of 60 μl per well, and incubated for a minimum of 1 h at 37°C.

#### Preparation of cortical cultures

Primary cortical cultures were established from E18 Sprague Dawley rats (*Rattus norvegicus*) using individuals of either sex (Charles River Laboratories, Crl:SD, RRID:RGD_737891). Dams were killed by CO_2_ euthanasia under a protocol approved by IACUC of Brigham and Women’s Hospital. Dissection of complete cortex from pups was performed in ice-cold HBSS (Gibco) under a dissecting microscope (Zeiss). The dissected cortices were suspended in 0.25% trypsin-EDTA (Gibco) for 10 min at 37°C, the excess trypsin-EDTA solution was then aspirated. The tissue was then triturated in Complete DMEM [DMEM culture media (Gibco) supplement with 5% fetal calf serum (Lonza) and 1× penicillin/streptomycin/L-glutamine (Gibco)] with a 10-ml serological pipette before being passed through a 100-μm strainer (BD Falcon). Counts of the cell suspension were taken in triplicate, and the cell suspension was back diluted to 1.5 × 10^6^ cells/ml in Complete DMEM. The cell suspension was added to the MEA plates in a volume of 50 μl for a plating concentration of 7.5 × 10^4^ cells/well or 2.4 × 10^5^ cells/cm^2^. Plates were then placed in a tissue culture incubator (37°C, 95% humidity, 5% CO_2_) for 4 h to allow for cells to attach to the culture surface. After the 4-h incubation, 150 μl of BrainPhys Media (1× BrainPhys culture media containing SM-1 neuronal supplement; StemCell Technologies) was added to each well. The cultures were maintained in a tissue culture incubator in BrainPhys Media, with semi-weekly half-volume media changes.

### Cell viability assay

Cell viability was assessed using the Pierce LDH Cytotoxicity assay kit (ThermoFisher Scientific) according to manufacturer’s protocol. Briefly, 50 μl of treated culture media along with 50 μl of detection reagent were combined in a clear 96-well microtitre plate, and incubated at room temperature for 30 min. Following incubation, the stop reagent was added, and optical density (OD) was measure at 490 and 680 nm using a Synergy HT plate reader (BioTek). Percentage viability was calculated by first subtracting the OD_680_ from the OD_490nm_, then normalizing each observation to the median values of the cell lysis positive control (PC) and the untreated negative control (NC) using the equation:

$$N(x)=(1-\frac{OD_{x}-\overset{∼}{OD_{NC}}}{\overset{∼}{OD_{PC}}-\overset{∼}{OD_{NC}}})\times 100$$

### MEA recording and analysis

All MEA recordings were performed using a Maestro multi-well MEA recorder (Axion Biosystems). During recordings, plates were kept on a heated stage maintained at 37°C and ventilated with a mixture of 5% CO_2_:95% air (AirGas) at a rate of 1 cubic-foot/h. to prevent evaporation of liquid within wells by convection and condensation on the underside of the plate lid, the MEA plate was covered with an air-activated oxidizing iron heater (HotHands, Kobayashi) placed on top of an aluminum plate cut to size, for even dispersal of heat. Voltage potentials within wells were simultaneously recorded across 768 channels (eight electrodes per well, 96-well plate) at a sampling frequency of 12.5 kHz using AxIS acquisition software version 2.4 (Axion Biosystems). This sampling rate of 12.5 kHz was chosen as it represents the maximum rate of the Axion Maestro instrument used. Note that sampling rates upwards of 20–50 kHZ are achievable with other commercially available multi-well MEA systems. The raw voltage recordings were subjected to a Butterworth filter of 200 Hz to 2.5 kHz, and neuronal firing events (spikes) were detected when the voltage exceeded a “crossing threshold” set at 5.5 SD away from the root mean square (RMS) of the background potential calculated over a 10-ms moving window. All recordings were performed for 30 min unless otherwise specified.

Raw voltage, timestamp, value of crossing threshold for each spike event were extracted from the.spk files of MEA recordings produced AxIS acquisition software, using custom MATLAB scripts (MathWorks) using extractor functions provided with AxIS version 2.4. Following extraction of the raw recording data, all analyses and simulations were performed using the R statistical programming language (R Core Team, 2017).

#### Mean firing rate (MFR) calculation

To remove spurious spike events arising from by “high-noise” electrodes, an upper limit to the crossing threshold was established by examining the crossing threshold (μV) for all spike events detected and calculated the value corresponding to 3 SD greater than the mean crossing threshold, all events detected at a crossing threshold greater than this upper limit were excluded from the analysis. The MFR (Hz) was calculated as the ratio of the total number of spikes recorded (*n*), and the duration of recording in seconds, $MFR=\frac{n}{s}$. The log transform of the MFR was calculated as $log_{10}(\frac{n+1}{s})$, to account for instances of *n* = 0, the log of which is undefined.

#### Treatment group assignment

A pool of active arrays from a multi-well MEAs was established by selecting those arrays that are no >2 SD below the median firing frequency (*log*_10_Hz) of the sample set. A panel of *i* possible treatment assignments is generated by randomly assigning arrays to treatment groups *g*, each with *n* members. For the purposes of this study, *i* = 10^4^. For each instance of *i*, a one-way ANOVA was performed, assessing *log*_10_Hz as a function of *g*. The instance of *i* resulting in the lowest value of the F-statistic, was used as the treatment group assignment.

#### Neurotoxin treatments

*α*-Hemolysin (*α*HL) and tetrodotoxin (TTX) were purchased from Sigma-Aldrich. Both reagents were reconstituted in sterile water, and diluted to the specified concentration in BrainPhys culture media containing SM-1 neuronal supplement (StemCell Technologies); 30-min baseline recordings of mature [more than day *in vitro* (DIV)21] rat primary cortical cultures were performed, and arrays were assigned to cohorts with comparable activity (six to eight replicates per group) using the technique described above. Cultures were treated with a titration of TTX (0.001–1 μM), and recorded again for 30 min both immediately following treatment and 24 h following treatment. For array-level spike frequency analysis the magnitude of effect of each condition was determined from the coefficients of a linear mix-effect model, while for cluster-level spike frequency analysis, the magnitude of effect of each condition was determined from the coefficients of Γ-distributed generalized linear model (GLM).

#### Spike sorting

For each spike event, the following metrics were calculated based on the vector of 38 momentary voltage measurements within each instance: maximum voltage (peak), minimum voltage (valley), wave form amplitude (peak-valley range), time interval between peak and valley, area under the curve (AUC), and non-linear energy (NLE). AUC was calculated using the auc function within the R MESS library (Ekstrøm, 2018), NLE was calculated using the equation provided by Kim and Kim (2000):

$$\Psi (x_{n})=x_{n}^{2}-(x_{n-1}\times x_{n+1}).$$

The data were aggregated across all recordings in a given experiment, and segmented based on individual electrode. Principal components analysis was performed using the prcomp function within the base R stats library, following log transformation of the metrics as recommended by Venables and Ripley (2002). Mean-shift clustering of spike events was performed based on the values of the first two principal components using the msClustering function within the R MeanShift library (Ciollaro and Wang, 2016), using a Gaussian kernel and bandwidth value of *h* = 1.5.

#### Network analysis

Spike clusters identified by unsupervised spike sorting analysis were deemed to represent individual neurons. Functional connections between neurons were established based on the spike time tiling coefficient (STTC) calculated between the spike trains of events attributed to each individual. STTC was calculated by the method reported by Cutts and Eglen (2014). Where between two spike trains *A* and *B*,

$$STTC=\frac{1}{2}\left(\frac{P_{A}-T_{B}}{1-P_{A}T_{B}}+\frac{P_{B}-T_{A}}{1-P_{B}T_{A}}\right),$$

with a Δ *t* value of 100 ms. Observed STTC values were taken to represent functional connections if they below the 0.05% or above the 99.5% quantile of a null distribution STTC values derived from 1000 permutations of random spike trains with comparable numbers of events as *A* and *B*. The network cluster coefficient ($\bar{C}$) as reported by Watts and Strogatz (1998) was calculated within each array and recording using the using the graph from data frame and transitivity functions within the R igraph library (Csardi and Nepusz, 2006), with isolates treated as zeros.

All spike sorting, permutation, and simulation analyses were performed on the O_2_ high performance computing cluster (Research Computing Group, Harvard Medical School). All scripts for performing these analyses are available at https://github.com/SubstantiaNegri/meaAnalysis.

### Statistical analyses

#### Power calculation

A dataset of 30-min recordings of 1272 unique, untreated MEAs was taken to represent spontaneous firing activity *log*_10_Hz at time (*t*_0_), this was defined as vector *A*. The correlation coefficient between the firing frequencies of a population MEAs recorded at separate times was estimated to be *ρ* = 0.8, based on the repeated recordings of multiple culture preparations at DIV20 and DIV21. To simulate the expected variance in firing frequencies between recordings, a second vector *B* was calculated by the formula:

$$B=\rho A\;+\;A^{⊥}\sqrt{\;1\;-\;\rho^{2}},$$

where $A^{⊥}$ represents the residuals of a linear regression between *A* and a sample of equal length drawn from a random normal distribution. The resulting value of *B* was examined to confirm that:

$$cor(\vec{A},\vec{B})=\rho ,$$

*B* is then taken to represent spontaneous firing activity *log*_10_Hz at time (*t*_1_). For each simulated experiment, *control* and *treatment* groups were generated by drawing the paired *log*_10_Hz*_t_*_0_ and *log*_10_Hz*_t_*_1_ values for random arrays for sample sizes ranging 3–16. The *log*_10_Hz*_t_*_1_ values within the treatment group were offset by effect sizes ranging from 0.1 to 2.0 *log*_10_Hz. A total of 5000 iterations were performed for each sample size:effect size pairing, for a total of 1.4 × 10^6^ total simulated treatments. For each iteration, an analysis of covariance (ANCOVA) was performed by fitting an ANOVA model to linear regression for *log*_10_Hz as a function of group (control/treatment) and time (pre/post) allowing for interaction between the group and time variables. The coefficients for the model were compared using Tukey’s test for honest significant difference, and the *p* value for the comparison of control versus treatment at time *t*_1_ was extracted. The linear regression, fitting of the ANOVA, and Tukey’s test were performed using the lm, aov, and TukeyHSD functions within the base R stats library. Power was calculated as the proportion of iterations within each sample size:effect size pairing for which the difference between control and treatment was calculated to have a *p* < 0.05.

#### Γ-distributed GLMs (Γ-GLMs)

For the distribution of spike cluster frequencies, Γ-GLMs were constructed using the glm function within the base R stats library. Firing frequency, Hz, was modeled as the dependent variable, while treatment and recording were used as interacting categorical independent variables. The inverse link function was applied for translating the model coefficients estimating *β*, the rate term of the Γ distribution, to the distribution average *μ*.

#### Linear mixed effect models

For estimating changes in array-level spike frequency and cluster-level wave form, amplitudes were constructed using the lme function within the R nlme library (Pinheiro et al., 2018). In all cases, the magnitude of effect of each condition was determined by aggregating the data from a minimum of three separate experiments. For array-level spike frequency, *log*_10_*Hz* was modeled as the dependent variable; treatment and recording (pre-treatment, post-treatment) were interacting categorical fix-effect variables; and each individual experiment as a random effect. For cluster-level wave form amplitude, *log*_10_μV was modeled as the dependent variable; treatment and recording were interacting categorical fix-effect variables; and spike cluster, electrode, and experiment were nested random-effect variables.

#### General linear hypothesis tests

General linear hypothesis tests for comparing the predictions of the GLM and LMEs were performed using the contrMat, glht summary.glht functions within the R multcomp library (Hothorn et al., 2008). Adjustments to p.values for multiple comparisons was done using the default “single-step” method within summary.glht. All figures were generated using the R ggplot2 and accompanying ggpubr libraries (Wickham, 2016; Kassambara, 2018).

## Results

### Prior art of MEA methodologies

As with all bio-assays, there are several facets to consider in experimental design and analysis. While many are universal, some are specific to the particular mode of detection. Those most relevant to the use of MEAs are detailed in Table 1. to assess existing practices for these elements of MEA experiments, the methods employed in 22 reported studies were reviewed. While not all of the experimental design elements list in Table 1 were explicitly addressed among the reported methods of these studies, the most commonly documented aspects of the methodologies are shown in Table 2.

**Table 1.** Considerations for MEA experimental design

Experimental conditions	Analysis parameters
Array surface preparation	Spike detection/event filtering
Culture media	Assay metric
Age of cultures	Array exclusion criteria
Recording duration	Treatment group assignment
Environmental control	Statistical analysis

**Table 2.** Comparison of MEA methodology

Reference	Instrument format	Culture model	Surface preparation	Culture media	Age (DIV)	Duration (min)	Detection threshold	Assay metric(s)	Statistical analysis
Varghese et al. (2010)	Single-well	Primary rat (E18)	DETA	NB	12–16	-	-	MFR	*t* test
Kuperstein et al. (2010)	Single-well	Primary mouse	-	-	8–10	45	5 SD	MFR	*t* test
McConnell et al. (2012)	Multi-well	Primary rat (P2)	Laminin	NB	12–22	33	8 SD	MFR, active channels	-
Frega et al. (2012)	Single-well	Primary rat (E18)	PDL+laminin	NB	21–28	20	-	MFR, burst rate	Kruskal–Wallis
Bateup et al. (2013a)	Single-well	Primary mouse	PDL+laminin	NB	-	2	9 μV	MFR	*t* test, ANOVA
Biffi et al. (2013)	Single-well	Primary mouse	PLL	NB	4–35	10	5X baseline	MFR, active channels	Kruskal–Wallis
Vincent et al. (2013)	Single-well	Primary rat (E18)	PEI	-	-	20	3 SD	MFR	Wilcoxon
Bateup et al. (2013b)	Single-well	Primary mouse	PDL+laminin	-	12–19	2	9 μV	MFR	*t* test, ANOVA
Wainger et al. (2015)	Single-well	Reprogram-fibroblast neurons	PDL+laminin	DMEM/F12	>28	-	10 μV	MFR	Mann–Whitney
Wainger et al. (2014)	Both	hIPS-derived neurons	PDL+laminin	-	>24	-	10 μV, 5.5 SD	MFR	Kruskal–Wallis, linear regression
Illes et al. (2014)	Single-well	Primary mouse	PDL+laminin	DMEM/F12	>21	-	6.2 SD of baseline	MFR	*t* test, ANOVA
Weir et al. (2014)	Single-well	Primary mouse (P1)	-	aCSF	7–21	-	7SD	MFR, burst rate, etc.	*t* test, ANOVA
Charkhkar et al. (2015)	Single-well	Primary mouse (E17)	PDL+laminin	DMEM	21	20	5 SD	MFR	*t* test, ANOVA
Vertkin et al. (2015)	Single-well	Primary mouse (P2)	-	-	14–21	-	-	MFR	*t* test, ANOVA
Bardy et al. (2015)	Multi-well	hIPS-derived neurons	Laminin	BP	2–21	10	6 SD	MFR	-
Slomowitz et al. (2015)	Single-well	Primary mouse (P2)	-	-	15–22	60	4–5 SD	MFR	-
Liu et al. (2017)	Single-well	Primary mouse	-	-	-	15	6 SD	MFR	*t* test
Alshawaf et al. (2018)	Single-well	hESC-derived neurons	-	NB	12–56	5	5 SD	MFR, active channels	Kruskal–Wallis
Feng et al. (2018)	Multi-well	Primary mouse (P1)	PEI+laminin	NB	7–28	30	-	MFR, burst rate, etc.	*t* test
García-León et al. (2018)	Multi-well	hIPS-derived neurons	PEI+laminin	DMEM/F12	70	5	-	MFR, burst rate, etc.	*t* test
Black et al. (2018)	Multi-well	Primary adult mouse DRG	PEI+PDL+laminin	DMEM/F12	3–21	30	5.5 SD	MFR, active channels	Mann–Whitney
Nehme et al. (2018)	Multi-well	hIPS-derived neurons	-	NB	7–42	-	5 SD	MFR, burst rate, etc.	Kruskal–Wallis
DeRosa et al. (2018)	Multi-well	hIPS-derived neurons	PEI+laminin	NB	>30	10	5.25 SD	MFR	ANOVA
Sarkar et al. (2018)	Multi-well	hIPS-derived neurons	PO+laminin	DMEM/F12	>21	10	5.5–6 SD	MFR	*t* test, ANOVA
Russo et al. (2018)	Multi-well	hIPS-derived neurons	PO+laminin	NB	>30	3	5.5 SD	MFR	Mann–Whitney
Blake et al. (2018)	Single-well	Primary adult mouse DRG	PEI+laminin	NB	7	-	5.5 SD	MFR	*t* test, ANOVA

Surface preparation: DETA, diethylenetriamine; PDL, poly-D-lysine; PLL, poly-L-lysine; PEI, polyethylenimine; PO; polyornithine.

Culture media: NB, neurobasal media; DMEM/F12, Dulbecco’s modified eagle medium: nutrient mixture F-12; aCSF, artifical CSF; BP, BrainPhys media.

Vacancies in the table indicate instances where the reagent or technique used was not reported or unclear in the study methods.

### Calculation of MFR and spike detection threshold

Novellino et al. (2011) surveyed six toxicology laboratories employing MEAs; comparing methods of tissue culture, recording, signal processing and assay metrics across the different research groups. Additionally, the study had each laboratory test clinically used neuropharmacological agents in their assays to assess the reproducibility of the results. Based on the parallel testing of pharmacological agents, Novellino et al., found that MFR, defined as the ratio of total spike events to recording duration in seconds, was the most consistent metric of activity. Concordant with this report, the use of MFR for reporting spike frequency within MEA recordings was the most common assay metric reported by the other studies examined (Table 2).

**Figure 1.**
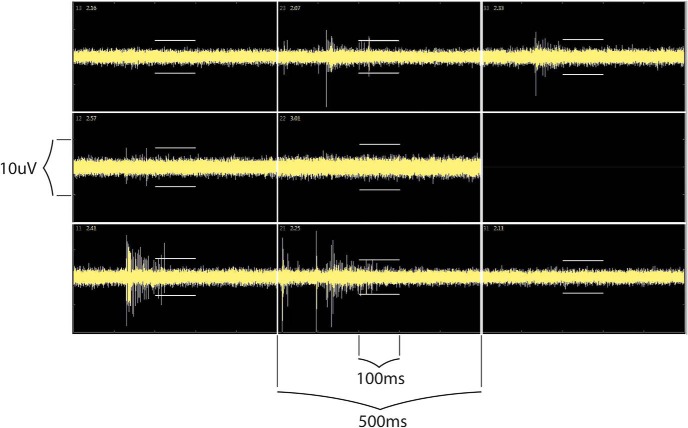
Example of MEA recording traces raw voltage recording of single well from a 96-well MEA containing eight electrodes. Each panel depicts 500 ms of recording data. The crossing thresholds for spike detection representing 5.5 SD the root mean square (RMS) of background voltage indicated by white bars above and below signal traces. Recording captured using AxIS acquistion software, Axion BioSystems.

The calculation of MFR is dependent on the detection of spontaneously generated action potentials, spikes, which occurs when the recorded voltage exceeds a designated threshold, as shown in Figure 1. Among the reported studies, this threshold was either set as an absolute potential value (Bateup et al., 2013a; Wainger et al., 2014) or relative to a number of SDs above the root mean square of the background voltage. In the latter case, the threshold determined by *n* SD can be dictated by an initial baseline recording (as in Illes et al., 2014). However, a method of dynamic threshold calculation over a moving window (in the range of ∼10 ms) was more common. The crossing threshold employed by most studies were in the range of 5–6 SD, although others have used crossing thresholds as low as 3 SD (Vincent et al., 2013) or as high as 8 SD (McConnell et al., 2012). While methods are available to detect subthreshold potentials within MEA recording (Henningson and Illes, 2017), the use of large magnitude thresholds is done to insure that the majority of the events recorded represent action potentials.

While any of these approaches for establishing a spike-detection threshold are valid, the dynamically calculated crossing threshold has the value of being extendable for use in a *post hoc* analysis to eliminate spike calls arising from spurious electrical noise. During recording, sporadic electrical noise can cause the background potential to increase several fold, resulting in the erroneous determination of these events as spiking events. This can occur while using a fixed or dynamically calculated threshold, since while the dynamic threshold will eventually adjust there is latency in doing so. Further, due to the hundreds of channels being recorded for tens of minutes or more, the probability of this occurring is increased, and manual supervision impractical. While this is not discussed in the reviewed literature, to address this issue as part of recordings performed for this study, the values of the crossing threshold at the time of the spike detection for all instances within a recording were examined. Since the background potential is expected to be random normal, all spikes for which the value of the crossing threshold at time of detection exceeded 3 SD of distribution across all crossing thresholds were excluded from analysis. This conservative threshold was chosen as to only exclude events from those channels exhibiting highly deviate background potentials. Following the removal of spike events detected at these aberrantly high crossing thresholds, the MFR was calculated as described above integrating all spike events recorded across all electrodes on an array during a recording session.

### Firing frequency from neuronal arrays exhibits log-normal distribution

**Figure 2.**
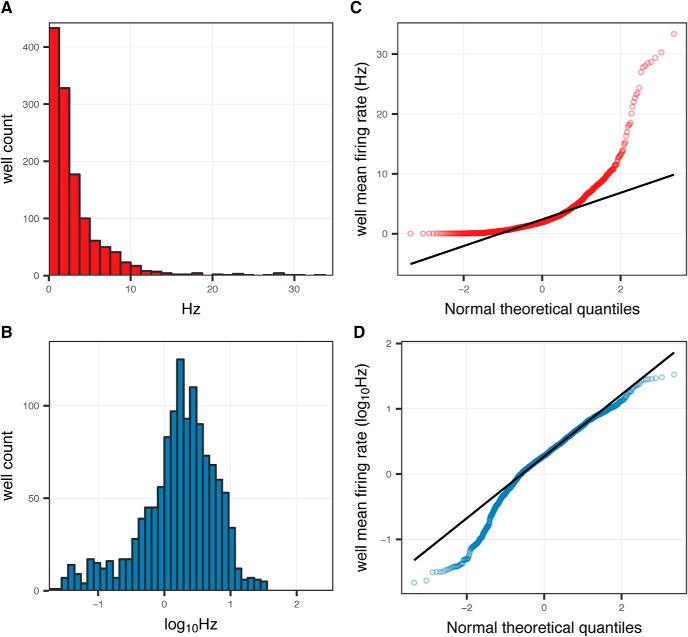
Distribution of MFRs observed in primary cortical cultures. Left, Distribution of firing frequencies (Hz) from 1272 unique arrays across 35, 96-well MEA plates originating from 22 separate culture preparations. Cultures were between DIV19 and DIV35 and recorded for 30 min. MFR shown on a linear (***A***, red) or log10-transformed (***B***, blue) scale. Arrays exhibiting low firing frequencies, MFR < 0.02 Hz have been removed from this dataset. Quartile-quartile plots comparing firing frequencies on a linear (***C***), log scale (***D***) to a random normal distribution (line).

To assess the distribution of firing frequencies observed across neuronal cultures on MEAs, the MFRs of untreated rat primary cortical cultures were examined (Fig. 2). In Figure 2*A*, the histogram shows firing frequencies reveals that the distribution is highly skewed on linear scale of Hz. Most studies reporting on MEA experiments make no mention of this highly skewed distribution in firing rates, with the noted exception of the report by Biffi and colleagues that discusses it specifically (Biffi et al., 2013). This observation may have gone unnoticed, due to studies being performed in single-well MEAs having insufficient data for this distribution to be apparent, however a similar highly skewed distribution has been reported for firing frequencies at individual recording electrodes within MEAs (Vincent et al., 2013). This distribution shape is not surprising, given that any frequency based metric is lower bound at zero, and the only upper limit is the sampling rate at which the observations are being made.

A practical consequence of this pattern of behavior is that the observed distribution array firing frequencies and a theoretical random normal distribution deviate substantially from each other as shown in Figure 2*C*. Despite this, many studies reporting on spontaneous firing activity of neuronal cultures recorded by MEAs use common parametric statistical tests such as Student’s *t* test and ANOVA, which assume a normally distributed dependent variable. Other researchers have presumably observed the asymmetry in the data sets of MEA recordings, and instead have used non-parametric tests such as Kruskal–Wallis and Mann–Whitney *U* tests for reporting their results (Table 2).

The distribution of array firing frequencies does form a symmetrical, approximately normal distribution following transformation of the values by *log*_10_, as shown in Figure 2*B*. Other studies have used *log*_10_ transformation in the reporting of MEA data, including Wainger et al. (2014) and Black et al. (2018), who did so to compare firing frequencies of individual electrodes, as well as Slomowitz et al. (2015), to compare the activity of individual neurons following spike sorting. Given the log-normal behavior of the observed frequencies, *log*_10_ Hz rather than Hz appears to be a more appropriate metric to describe the spontaneous firing activity of neuronal cultures, especially to enable analysis using parametric methods.

### Duration of recording for achieving intra-array reproducibility

Given the broad range of firing activity observed across arrays, extending more than two orders of magnitude, an important consideration for MEA based experiments is the duration of recording to perform. While the inter-array variance is the accumulation of the implicit variability of the biological system, and any variability introduced by the construction of the experimental system, the goal is to minimize sampling error by identifying a duration of recording sufficient to accurately capture the activity across the cultures. Most studies employing MEAs report using recordings on the order of tens of minutes (Table 2), although the duration can vary dramatically with some studies performing recordings as short as 2 min (Bateup et al., 2013a) and others up to 1 h (Slomowitz et al., 2015).

**Figure 3.**
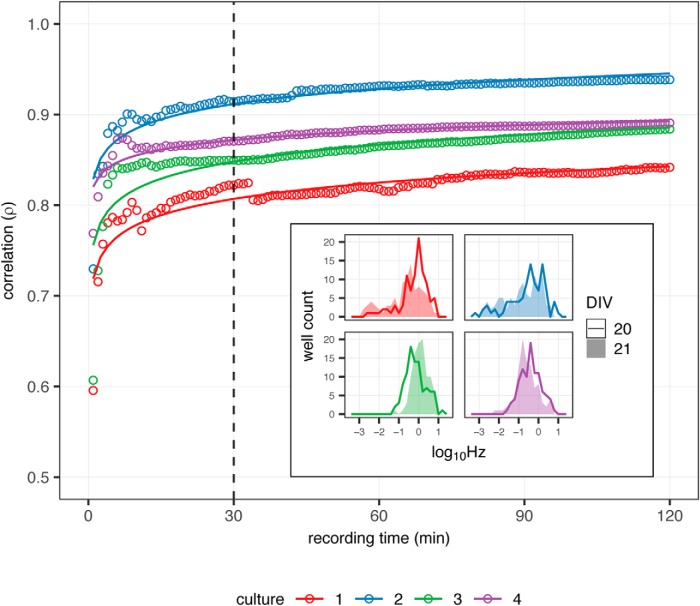
Correlation of firing frequencies in repeated recording as a function of time. Pearson’s correlation *ρ* between firing frequencies of arrays on consecutive days (DIV20 and DIV21) as function of recording time for four culture preparations. Line indicates fit of *ρ* ∝ ln(time). Initial 2- of 3-h recording shown. Inset, Distribution of array firing frequencies *log*_10_Hz for 30 min during each recording.

To formally address this question of what is a sufficient duration of recording, the correlation of firing frequencies was examined as a function of recording length in repeated recording sessions. Extended 3-h recordings were taken of mature cortical cultures performed on consecutive days (DIV20, DIV21). From these data, the Pearson’s correlation (*ρ*) was calculated between the distribution of MFRs of arrays on the first day versus the second day, for accumulating 1-min intervals starting from the initiation of recording out to the entirety of the recording. The correlation of firing activity from recordings across sequential days increases dramatically within the first 30 min of recording with a value *ρ* > 0.8 observed in all experiments, yet there are only modest increases in *ρ* seen comparing longer intervals (Fig. 3). Fitting a linear regression to the data shows that the correlation between recordings is approximated by the natural log of recording duration ρ ∝ ln(time). This suggests that substantially increasing the length of recording beyond 30 min will only marginally improve the correlation, since $\frac{\delta }{\delta x}ln(x)=\frac{1}{x}$. Based on these observations, 30-min recordings appear to be an adequate recording interval to measure firing activity within MEAs for three-week rat cortical neuronal cultures. This method of analysis can be employed for any cellular system to determine the most appropriate recording time for the culture conditions to be used in a study using MEAs.

### Changes in spontaneous firing across arrays with culture maturity

From the published literature, studies performing MEA experiments using primary cultures of rodent cortical neurons have a range of reported age of cultures: DIV post plating from dissected brain, and the time of analysis ranges from DIV7 to DIV35, although DIV14–DIV28 is most common (Table 2). Conversely, studies that instead used neuronal cultures derived from stem cells have a range of culture ages differing to a greater degree, over 30 d (DeRosa et al., 2018; Russo et al., 2018), including up to DIV70 (García-León et al., 2018). This broad range is expected given the variety of differentiation protocols used and increased interval until these cells become electrically active.

**Figure 4.**
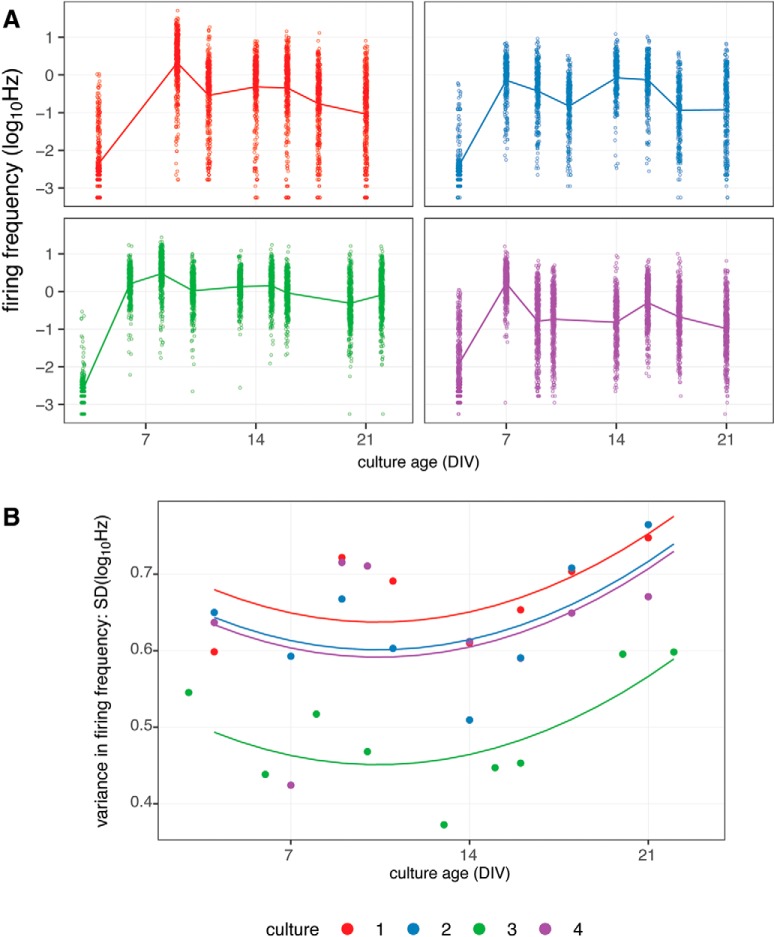
Changes in firing frequency with culture maturation. ***A***, Firing frequencies of arrays observed during repeated 30-min recordings over a three-week period (DIV3–DIV22) across four culture preparations. Each point represents an individual array per day; 384 replicate arrays, cultures: 1, 3, and 4; 288 culture: 2. Line indicates mean logHz per recording for each culture. ***B***, SD in firing frequencies of arrays observed during repeated 30-min recordings shown in panel ***A***. Each line represents the prediction of a linear model for the change in variance as a function of time.

To assess how firing activity changed with maturation, four culture preparations were recorded every 2–3 d between ages of DIV3 and DIV22. As shown in Figure 4*A*, the cultures are electrically active by the end of the first week, and that activity remains generally consistent over the next two weeks. Of course, as described above, the range of activity observed across the population of arrays is broad. Additionally, this depiction shows all arrays, including the portion from which little electrical activity is observed. These are apparent in the lower portion of the figure where the data appears striated, where differences in a single spike event are seen as step-wise changes.

To determine whether the distribution of firing frequencies differs between cultures, further analysis was performed to examine the variance of firing frequencies with culture maturation (Fig. 4*B*). ANOVA indicates that the SD of firing frequencies is significantly affected by culture preparation and the age of the culture. Comparison of models indicates that the variance is significantly affected by culture age when controlling for the effect of culture preparation (*p* = 0.013). Examining the effect of culture preparation indicates that culture 3 (green data points) does show significantly lower variance than that of cultures 1, 2, and 4 (*p* < 0.001, *p* < 0.001, and *p* = 0.0014, respectively). However, there is insufficient evidence to conclude interaction between culture preparation and culture age. Put more simply, the rate of change in variance across firing activity with respect to time appears consistent across cultures. The implications these data have for assay development using MEA platforms are that the variance across arrays within a single preparation of primary cortical cultures appear to reach a minimum between DIV7 and DIV14. However, substantial variation across array activity persists during this interval. Using cultures at this age may help mitigate some of the array-to-array variability, but other methods described in this paper such as the bootstrapping simulations for experimental group assignment will still be necessary to fully account for the observed variance.

### Assignment of treatment groups for MEA experiments

**Figure 5.**
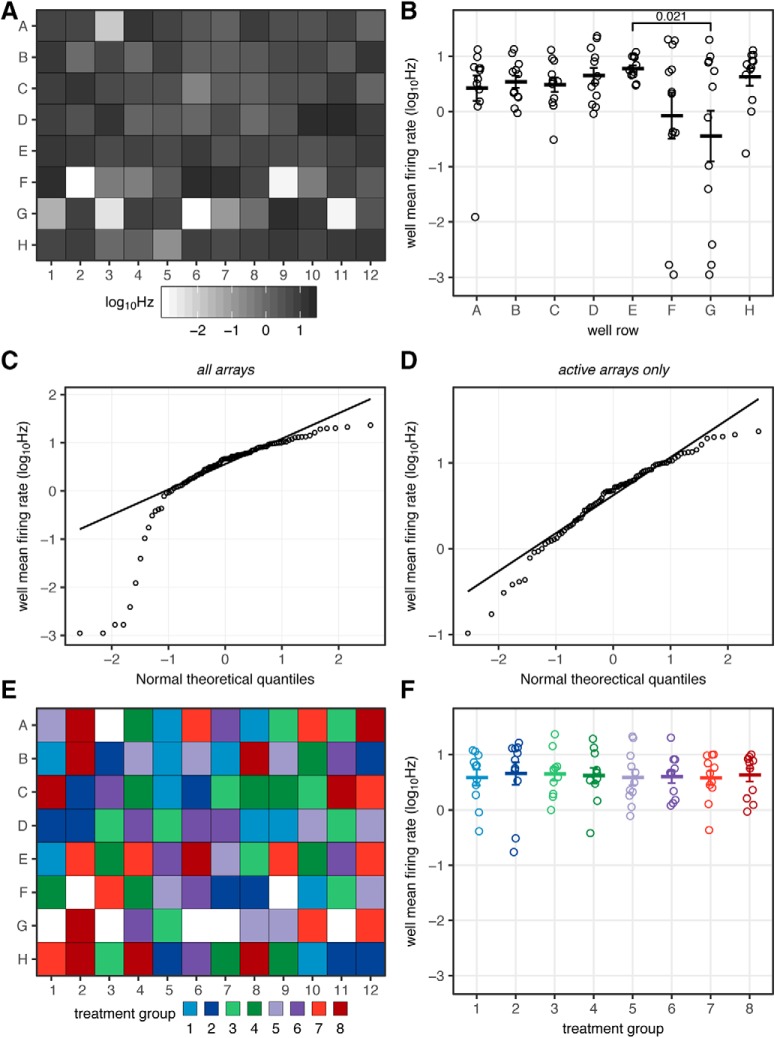
Assignment of treatment groups across multi-well MEAs. ***A***, Firing frequencies observed across a single recording of a 96-well MEA. ***B***, Firing frequencies across eight cohorts with 12 members (96 total), arising from cohort assignment based on plate row. QQ plots comparing firing frequencies to normal distribution, for all wells (***C***), or following exclusion of wells >2 SD below median (***D***). ***E***, Alternative assignment for eight cohorts with 11 members (88 total), with minimal between group variance, following simulation of 10,000 possible assignments. ***F***, Firing frequencies within groups from alternative group assignment. Data presented as mean ± SEM.

The variability observed across MEAs presents a challenge for the assignment of treatment groups for experiments. A common practice for biological assays within multi-well plates is to use the orientation of the wells in rows and columns to spatially assign treatment groups. However, this practice can be problematic for multi-well MEAs where variability is large and non-uniform. This is demonstrated for a typical multi-well MEA plate shown in Figure 5. Relying on plate rows for the designation of treatment groups can result in significant differences in the firing activity between cohorts even before the initiation of treatment, as shown in Figure 5*B*, where eight treatment groups (*n* = 12) were designated using the eight rows of the 96-well plate.

While plate-based variability does occur in conventional, colorimetric, fluorescence, or luminescence-based plate assays; in these instances, the cause is usually an external factor, such as liquid handling or excess evaporation along exterior wells, and are typically uniform plate-to-plate. Therefore, these can be addressed by changes to the assay protocol, or *post hoc* application of statistical regression techniques (Clemons et al., 2009). However, unlike in many bioassays data acquisition of MEAs is non-destructive to the cultures, so the varying levels of activity between arrays can be assessed before initiation of experimental treatments.

The first step in remediating the issue posed by variability across MEAs is to exclude those arrays that exhibit substantially lower levels of firing activity than the population as a whole. These wells are clearly distinguishable as deviating from the otherwise normally distributed population (Fig. 5*C*). In practice, these low-activity wells are excluded by setting a threshold at 2 SD below the median value of firing frequency across all arrays in the experiment. In this case, seven arrays of 96 are excluded with the remainder closely following a normal distribution (Fig. 5*D*). Having eliminated these arrays, this now invalidates the approach of using plate rows or columns for the assignment of treatment groups, since the number of available replicates in each group would differ.

While aligning treatment groups to the rows and columns is convenient, it imposes an unnecessary constraint on the positioning of replicates within cohorts. For the original configuration of assigning 96 arrays to eight treatment groups with 12 members each, there are 6.25 × 10^14^ potential combinations, of which the option that aligns with the plate rows is only one. When the assay signal is uniform across the plate, one combination of wells is effectively as good as another, so opting for the most convenient makes the most sense. However, with the convenient option removed due to the exclusion of inactive arrays, and adding to it the non-uniform variability across the plate, there is reason to explore the other options to achieve a comparable level of pre-treatment activity between groups.

With the inactive arrays removed, the goal is to find an alternative assignment using the 88 most active of the remaining 89 arrays to generate 8 treatment groups with 11 members each. To do so, we have applied a bootstrapping simulation approach where 10^4^ possible treatment assignments are generated, then a one-way ANOVA is applied to each iteration assessing firing frequency (*log*_10_Hz) as a function of group assignment. From these ANOVA results, the iteration that results in the lowest F-statistic, which represents the ratio between group and within group variance, is selected for the assignment of the treatment groups. The results of this approach are shown in Figure 5*E*,*F*, where the alternative assignment results have near equal levels of activity across all eight cohorts. Using the described method of removing inactive arrays and determining the optimal assignment through simulation results in a 78.7% decrease in the variance in firing frequency associated with treatment group assignment (*TotalSS_sim_* = 17.64 vs *TotalSS_row_* = 82.6).

Additionally, this method can be extended to generate treatment groups with comparable activity levels across multiple plates for large experiments, which exceed the capacity of a single plate. It also can be used to generate treatment groups with comparable activity but uneven numbers of replicates for instances where the experiment incorporates a finite resource, like primary patient material, necessitating an unbalanced design.

### Adding it all up: power to detect changes in neuronal firing using MEAs

Ultimately, for a researcher seeking to model changes in spontaneous firing activity within neuronal cultures using MEAs, one of the most practical considerations of experimental design comes down to the decision of the number of replicates per condition. Having characterized the distribution of firing frequencies observed across a large number of MEAs (Fig. 2) and having established the correlation of activity between repeated recordings (Fig. 3), it is possible to use these factors to estimate the statistical power of MEA experiments.

For demonstration purposes, a two condition (control, treatment), repeated measures (pre, post), experiment is modeled. A bootstrapping approach is applied to simulate experiments using sample sizes ranging from three to 16 replicates per condition, and scenarios in which the treatment resulted in effect sizes of a difference in *log*_10_Hz ranging from 0.1 to 2.0. The dataset used for the simulation is generated from the firing frequencies of 1272 unique MEAs. The pre-treatment time point *log*_10_Hz*_t_*_0_, is taken from the existing data, while the post-treatment time point *log*_10_Hz*_t_*_1_ is randomly generated as to be related to *log*_10_Hz*_t_*_0_ by a correlation coefficient *ρ* = 0.8, estimated from repeated recordings of MEAs (Fig. 3). The firing frequencies of experimental replicates are pairs of *log*_10_Hz*_t_*_0_ and *log*_10_Hz*_t_*_01_ with values drawn from random arrays in the population. The *log*_10_Hz*_t_*_01_ values within the treatment condition are offset by the specified effect size. to estimate the difference between control and treatment at the post time point, and an ANCOVA is performed assigning time as a co-variate and determining the effect of treatment. This method has been shown to provide higher power than other used approaches, such as percentage of baseline or absolute change (Vickers, 2001).

**Figure 6.**
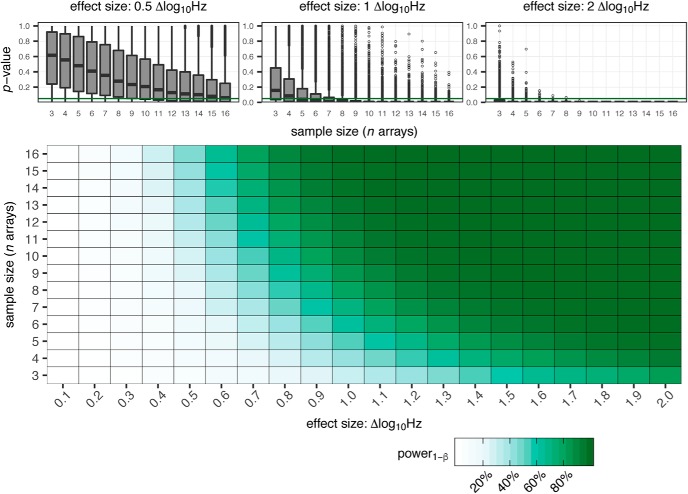
Calculation of statistical power for MEA experiments. Estimates of percentage power_(1-_*_β_*_)_ based on simulation for a two-condition (control, treatment) repeated measures (pre, post) experiment across sample sizes 3–16 arrays and effect sizes of 0.1–2.0 Δ *log*_10_Hz. Simulated experiments analyzed by ANCOVA, *p* value extracted from comparison between control and treatment at post measure, 5000 iterations per sample size:effect size pairing. Upper, Distributions of observed *p* values from simulated treatments per sample size for effect size of 0.5, 1.0, and 2.0 Δ *log*_10_Hz, green line indicates *p* = 0.05.

Applying the conventional standard of statistical power ≥80% for a robust assay, the results of this simulation show that it is difficult to reliably detect changes below 0.7 Δ *log*_10_Hz, ∼5 $\frac{spikes}{sec}$, even with large numbers of replicates. Based on the results of addition simulations, detecting changes on the order of 0.5 Δ *log*_10_Hz would require treatment groups with >40 replicates, and a change of 0.1 would require >600 replicates. to confidently detect changes of 1.0 Δ *log*_10_Hz (i.e., a 10-fold difference in $\frac{spikes}{sec}$), requires treatment groups with eight to nine replicates. Only with large changes in in firing frequency of Δ *log*_10_Hz 2.0 or greater (i.e., a 100-fold or more difference in $\frac{spikes}{sec}$) would it be reasonable to use treatment groups as small as an *n* = 3 (Figure 6). To illustrate the magnitude of these proposed effects, repeated experiments with TTX, showed that treating cultures with 10 nM induced decreases of –1.14 *log*_10_Hz and with 100 nM induced decreases of –2.24 *log*_10_Hz (data not shown). The reported IC_50_ of TTX-sensitive Na*_v_* channels in rats is 10 nM or less (Catterall et al., 2005). Therefore, this analysis suggests that to have sufficient power to detect changes in spontaneous firing activity comparable to those elicited by treatment with TTX at its IC_50_ concentration requires treatment groups with eight to nine arrays per condition. Of course, it is important to emphasize that these power estimates are based on a simple two condition experiment, so any experiment seeking to examine more conditions would need to allow for more replicates per condition to provide sufficient power for multiple comparisons. This estimate of a requirement of eight to 10 replicates to discern effects on firing frequencies within MEA between treatment conditions of strong perturbations is consistent with replicates used in studies reporting effect of known pharmaceutical compounds or neurodegenerative disease associated mutations (Novellino et al., 2011; Wainger et al., 2015).

### Implementation of unsupervised spike sorting for MEA recordings

MEA recordings provide a means of generating rich data sets of electrophysiological activity from a large number of neurons over comparatively long-time intervals of days to weeks. However, resolving the activity of individual neurons within this data are challenging due to the poor spatial resolution of MEA data acquisition since the density of detectors is sparse on a cellular scale, effectively rendering each electrode a point detector. Despite this, it is possible to deconvolute the mixed signals of multiple neurons detected on each recording electrode of a MEA through the implementation of spike sorting analysis in which shape parameters of waveforms arising from a population of neurons are binned by similarity allowing for attribution of individual waveforms to a cell of origin. However, results of spike sorting performed in this fashion come with the caveat that there is no “ground-truth,” since it is not possible to know exactly how many individuals neurons are being recorded and signals arising from separate cells may be conflated as coming from the same point of origin or vice versa due to fluctuations in field potentials and propagation of potentials across the array (for review, see Buzsáki et al., 2012; Gibson, 2012). The application of spike sorting analysis to MEA recordings is attractive as it allows for: (1) refinement of spike frequency metrics to be resolved to the level of individual neurons; (2) assessment of frequency-independent phenotypes, such as changes in the amplitude of potentials emitted from firing neurons; (3) determination of network dynamics within cultures through determination of coordinated firing patterns.

A review of commercially available software solutions capable of performing this analysis was performed. One such product available at the time of initiation of this study was Offline Sorter (Plexon) which has been used in the analysis of other MEA studies (Slomowitz et al., 2015; Vertkin et al., 2015). While this software has the capability to implement similar clustering algorithms to those ultimately employed in the analysis pipeline developed for this study, this software has critical limitations that limit its utility for the analysis of multi-well MEA recording data. First, this software is only capable of running on a single CPU, precluding the ability to take advantage of distributed computing resources available, and thus greatly increasing the processing times of analysis. Second, the software required the data to be analyzed in a recording centric fashion, which greatly diminishes the accuracy of the clustering.

The development criteria for the analysis pipeline were: (1) flexibility to analyze data collected during separate recording sessions; (2) ability to perform clustering analysis in an unsupervised fashion; (3) extendable to parallel processing in a distributed computing environment such as high-performance computing clusters; and (4) able to use open-source software to enable distribution and use by other researchers.

For these analyses, the recording sampling rate was 12.5 kHz, and the spike detection threshold was set at 5.5 SD from the root-mean square of the background voltage potential calculated over a 10-ms moving window. On recording of a voltage above the detection threshold (spike event), 3 ms of recording data were retained, 1 ms preceding and 2 ms following the timing of the threshold crossing event. With a sampling rate of 12.5 kHz, the 3-ms window around each threshold event results in the retention of 38 momentary voltage recordings for each spike event. These 38 momentary voltage recordings are reconstructed into a wave form. From the reconstructed wave form, an array of shape parameters are calculated for each event, including: maximum voltage (peak), minimum voltage (valley), wave form amplitude (peak-valley range), time interval between peak and valley, AUC, and NLE, a parameter describing the “sharpness” around the maxima and minima of the wave form (Kim and Kim, 2000).

**Figure 7.**
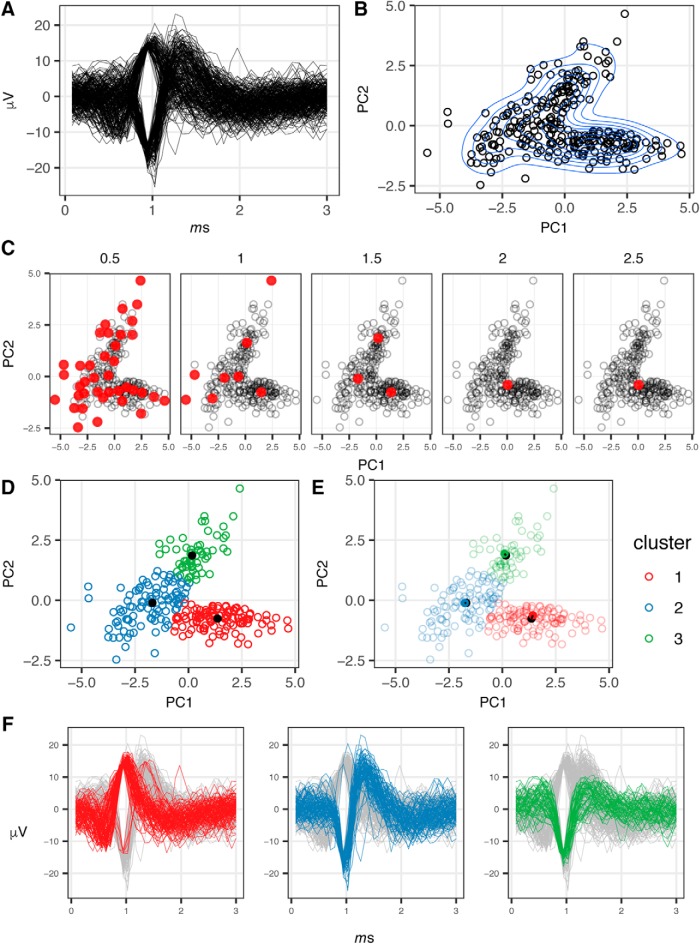
Demonstration of spike sorting. ***A***, Waveforms of 269 spike events captured by a single electrode across 7, 30-min recordings. ***B***, Projection of 269 spike events along principle first two principal components calculated from wave form shape parameters, blue lines indicate density contours of events. ***C***, Varying number of cluster centroids identified by mean-shift clustering as a function of bandwidth value *h* of KDE. Cluster centroids indicated in red, value of kernel density estimate denoted above each panel. ***D***, Spike events colored by cluster membership as determined by mean-shift clustering performed with a KDE *h* = 1.5, cluster centroids are indicated in black. ***E***, Mapping of cluster centroids (black circle) to nearest spike (filled circle) event by minimum Euclidean distance. ***F***, Waveforms of spike events colored by cluster membership.

The spike sorting process begins by aggregating all of the spike events detected by a given electrode during all recordings of an experiment, as shown in Figure 7*A*. Based on the wave form parameters of all spike events collected on a given electrode, principal component analysis (PCA) is performed and all spike events were projected along the first two principal components as shown in Figure 7*B*.

Once projected along principal components, clustering of the waveforms is performed to group those exhibiting the highest degree of similarity. to do so in an unsupervised fashion, the mean-shift algorithm was applied, implemented through use of the MeanShift library within the R programming language (Fukunaga and Hostetler, 1975; Comaniciu and Meer, 2002; Ciollaro and Wang, 2016). The use of mean-shift clustering has particular advantages for the analysis of MEA data over other non-hierarchical clustering techniques such as, *k*-means, since mean-shift does not require an a priori estimate of the number of groups in the dataset. This is an important practical consideration given that the number of cells that will be recorded on a single electrode is unknown and variable. While others have implemented semi-supervised clustering methods relying on *k*-means for the purpose of spike sorting in MEA recordings (Slomowitz et al., 2015), this requires manual intervention to indicate the number of distinct wave form shapes present on each channel. Instead, the mean-shift algorithm operates by applying a density function to the observed data and identifying the local maxima across the distribution of the observations, all observations within the dataset are then mapped or “shifted” to the nearest local maxima. The only variable that needs to be supplied to the algorithm is the bandwidth term (*h*) to the kernel density estimator (KDE) function to determine the degree of curvature supplied to the density function. For this analysis, a Gaussian density function was applied, while the value of the KDE was determined empirically by applying a range of values to sample clustering of a teach-set of electrodes to establish a value which provided accurate segregation of the waveforms, resulting in the use of *h* = 1.5. The influence of the KDE in the mean-shift algorithm is demonstrated in Figure 7*C*, in which lower values of *h* result in insufficient smoothing of the density function, resulting in an over proliferation of local maxima, while higher values of *h* result in over smoothing of the density function resulting in only a single maximum.

While the cluster centroids, as shown in Figure 7*D*, occupy a finite position with respect to the eigenvectors these points may or may not coincide with points representing individual, observed waveforms. Therefore to map the cluster centroid back to the original shape parameters used in deriving the clusters themselves, the wave form closest to the centroid is determined by calculating the minimum Euclidean distance (Fig. 7*D*,*E*). The shape parameters of the wave form closest to the centroid can thus be used for evaluating the mean shape differences between clusters.

### Spike sorting in a recording-dependent versus recording-independent fashion

**Figure 8.**
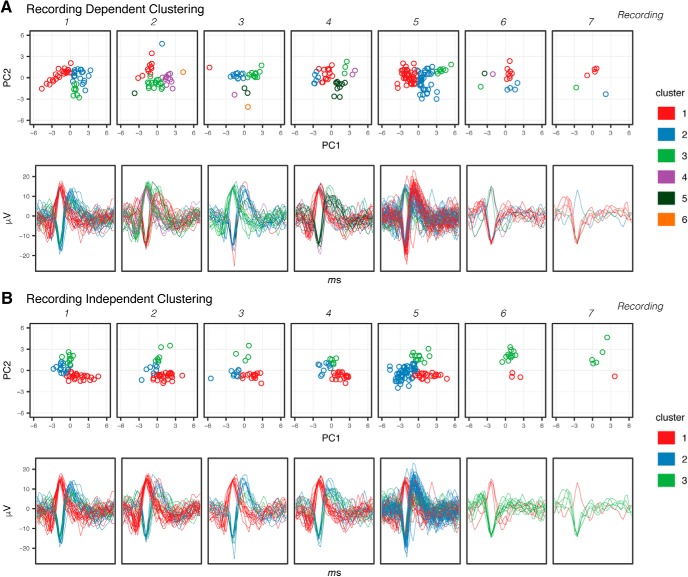
Spike sorting within single recordings versus independent of recording. Result of cluster assignment of 269 spike events recorded from a single electrode across 7, 30-min recordings. Mean-shift clustering performed in recording-dependent fashion on spike events from each recording (***A***) or in recording-independent fashion across pooled spike events from all recordings (***B***).

Given the longitudinal nature of many MEA-based experiments, it was important to assess the effect of performing the mean-shift clustering in a recording-dependent fashion, in which the clustering is performed several times, once for each set of single recording data, or in a recording-independent fashion, in which the clustering is performed once on the data from all recordings. In the latter case, the meta-data describing the recording origin of each event is maintained, so that the events can still be parsed by recording. The result of these two approaches is shown in Figure 8, in which spikes recorded by a single electrode across multiple recordings were clustered in a recording-dependent fashion (Fig. 8*A*) or recording-independent fashion (Fig. 8*B*). The limitation of the recording-depedent approach is exposed as a consequence of the uneven distribution of spike events across recordings. During the fifth recording in which 92 spikes are recorded, the recording-dependent method identifies the same number of clusters, three, as identified in the recording-independent analysis. However, during the third recording in which only 35 spikes are recorded, sporadic events that are otherwise aggregated into cluster #3 (green) in the recording-independent analysis are oversegregated in the clusters #4 (purple), #5 (dark green), #6 (orange) in the recording-dependent method. The other limitation to the recording-dependent approach is that since the values assigned denoting cluster membership are arbitrary, clusters that occupy the same position in principal component space are assigned to different cluster values in different recordings, presenting an additional challenge of correctly associating clusters hosting similarly shaped waveforms based on the cluster identifier alone.

These data emphasize the importance of performing spike-sorting in an recording-independent manner when examining recording data collected over several sessions to avoid misattribution of firing events to separate clusters that are in fact likely arising from a common point of origin.

### Stochastic nature of firing patterns of individual spike clusters

Having implemented spike sorting analysis, it is possible to evaluate the firing frequencies of individual spike clusters, providing a higher resolution of the spontaneous firing than obtained with the array (or well)-level as described above.

**Figure 9.**
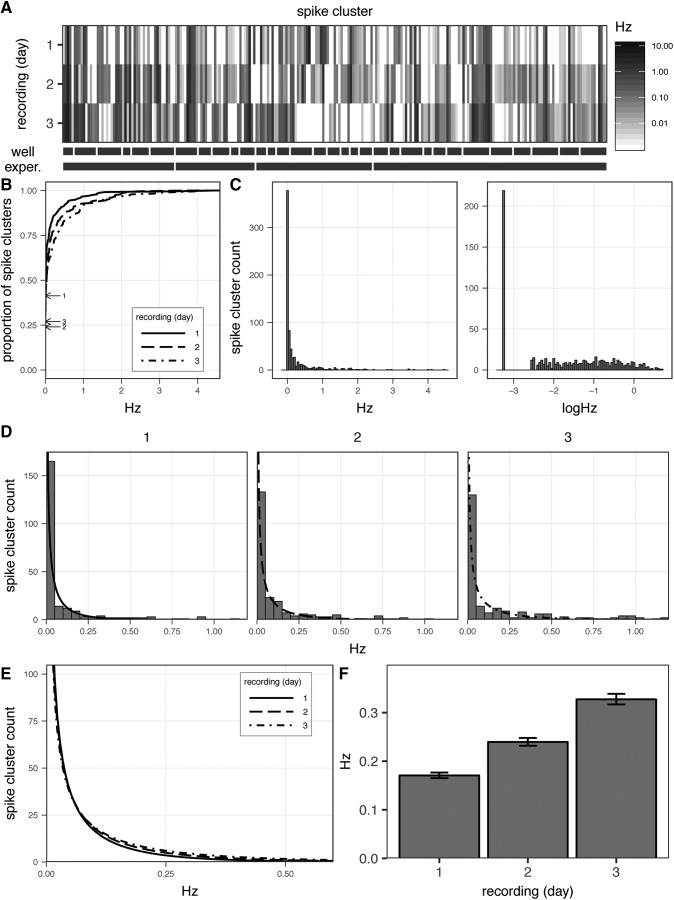
Stochastic firing patterns of spike clusters. ***A***, Firing frequencies (Hz) of 237 spike clusters identified in recordings of 156 electrodes in 32 replicate wells across 4 mature, untreated cultures (DIV20–DIV45). Data from 30-min recordings across three consecutive days. Spike clusters within common wells and experiments indicated by hash bars. ***B***, Cumulative distribution of spike cluster firing frequencies across recordings, arrows denote y-intercept, indicating proportion of inactive clusters during each recording. ***C***, Distribution of firing frequencies Hz across all three recordings on a linear (left) or log_10_-transformed scale (right). ***D***, Distribution of firing frequencies across each recording overlaid with estimated distribution of firing frequencies obtained from Γ-GLM. ***E***, Overlay comparing estimated distribution of firing frequencies from each recording. ***F***, Average firing frequency per recording estimated from Γ-GLM, presented as mean ± SE.

The firing frequencies of 237 spike clusters identified within 32 arrays wells across four untreated culture preparations, are shown in Figure 9*A*. These data show the firing frequencies as average $\frac{spikes}{sec}$ (Hz) from 30-min recordings taken on three consecutive days. Across the four experiments, the timing of culture media changes was coordinated with respect to the timing of recordings to minimize differences in firing across experiments as a consequence of the state of media exhaustion.

Examining firing frequencies at the level of individual spike clusters in Figure 9*A*, the patterns of firing over time appear largely stochastic. First, the firing frequencies, as indicated by increasing values from white → black of clusters differ dramatically with respect to each other across clusters within recordings (along rows) and within the same cluster across recordings (down columns), from a minimum of 0 Hz to a maximum of 15.96 Hz. Additionally, while all clusters are observed firing in at least one of the recordings, the number of instances in which clusters are inactive during any one 30-min recording interval in which the culture is observed is substantial. As shown in the cumulative density plot in Figure 9*B*, the proportion of inactive clusters during a given recording ranges from 24% to 41%. Additionally, while the majority of spike clusters exhibit firing frequencies are <1 Hz, there are a limited number of highly active clusters. The combination of a large proportion of inactive clusters and a limited number of highly active clusters results in a highly skewed distribution of firing frequencies (Fig. 9*C*, left) analogous to the distribution firing frequencies observed at the array-level (Fig. 2).

The zero-inflated nature of the cluster firing frequencies presents a challenge when aiming to describe differences in distributions of cluster frequencies, such as in response to experimental conditions. The strategy of applying a log transformation to the frequency values as described above for array-level frequency data are not applicable in this instance, since the result of the log transformation is not a symmetrical normal distribution as is achieved in that case, but rather an asymmetrical bi-modal distribution (Fig. 9*C*, right) However, since the values of the firing frequencies can theoretically assume values from 0 → acquisition sampling rate, an alternative approach is to model the distribution of cluster firing frequencies with a Γ distribution. While the distribution of non-transformed array-level firing frequencies could be described using a Γ distribution as well, in practice the log-transformation of array-level firing frequencies is more convenient since most arrays exhibit some basal level of activity across recordings compared to the large proportion of clusters which exhibit no activity during a given recording.

To assess changes in the firing frequencies across distributions of clusters under different experimental conditions, Γ-GLMs can be applied to obtain estimates for the average firing frequency in each condition (McCullagh and Nelder, 1989). This approach is demonstrated in Figure 9*D*, in which a Γ-GLM was fit to the data model frequency as a function of recording, and the resulting Γ distributions predicted by the model are overlaid on the distribution of clustering firing frequencies observed in each recording. The differences in firing frequencies across recordings are shown in Figure 9*E* comparing the Γ distributions predicted for each recording and in Figure 9*F* comparing the differences in average Hz ± SE between recordings based on the estimates obtained from the fitted model.

### Characterizing network behavior of cultures within MEA recordings

**Figure 10.**
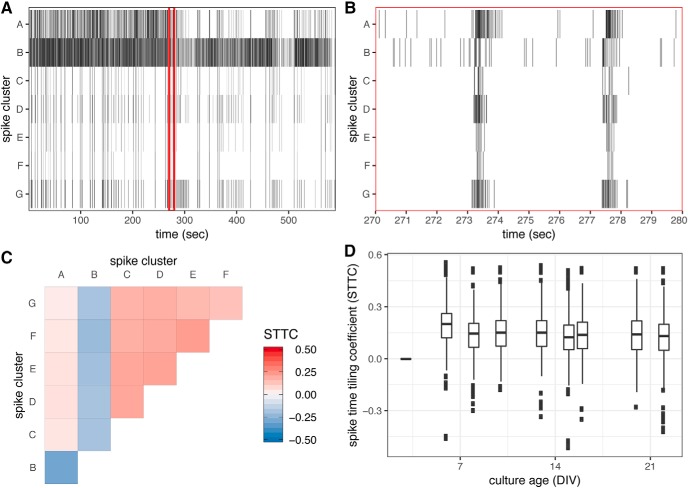
STTC derived from pairwise contemporaneous firing across spike clusters. ***A***, Raster plot indicating instances of action potentials attributed to seven individual neurons following unsupervised spike clustering of 10-min recording of DIV8 primary cortical culture. Red box indicates 10-s interval within the recording window. ***B***, Instances of action potentials within 10-s interval indicated within the longer 10-min recording depicted in panel ***A***. ***C***, Value of STTC calculated between spike clusters within a single array across a single 10-min recording. ***D***, Distribution of 42,486 STTC values calculated between 14,225 unique spike cluster pairings detected across 387 arrays recorded between DIV3 and DIV22.

Beyond quantifying the distributions of firing frequencies observed across individual neurons (spike clusters), having resolved the recording data to this level it is possible to examine the network dynamics within populations. Compiling all action potentials attributed to an individual neuron (spike cluster) through spike sorting over a given recording interval results in the formation of a spike train for each cell (Fig. 10). Examining the spike trains arising from neurons detected on a single array over an interval of minutes (Fig. 10*A*) reinforces the variability in firing frequencies described above. However, examining the time course of firing events over a duration of a few seconds reveals synchronized firing patterns (Fig. 10*B*), a phenotype of neuronal networks widely reported to be maintained in dissociated cultures (Segev et al., 2001; Van Pelt et al., 2004; Chen et al., 2006). The STTC is a parameter for describing the concordance or discordance of firing events between a pair of spike trains (Cutts and Eglen, 2014). Figure 10*C* shows the STTC between seven individuals detected within an array over a single 10-min recording. Examining the distribution of over 40,000 STTC values calculated from >14,000 unique pairs of neurons detected from repeated recordings of 387 arrays containing primary cortical cultures, shows that large magnitude, positive and negative, STTC values are detected within a week from initiation of culture (Fig. 10*D*). Moreover, while the distribution of observed STTC values are largely consistent and symmetrical over the observed period, the majority of values are positive indicative of a propensity for in-phase rather than out-of-phase activity relationships in this culture type.

Using STTC to assess coordinated activity between pairs of neurons, this can be extended to infer whether the two cells are functionally connected. Those bona fide connections thereby are considered to represent edges in a network of activity among the neurons. To determine which pairs of neurons are likely to be functionally connected a permutation based analysis was applied similar to that described by Vincent et al. (2013). For each pair of neurons, a null distribution of STTC values was generated by 1000 random permutations of the time stamps of the observed firing events. The empirically observed STTC values were compared to this random distribution, and only those instances in which the observed STTC value was of sufficiently strong correlation (positive or negative) that the likelihood of occurring by chance had a probability of <1% where taken to represent a functional connection between neurons. Using this method, the firing patterns from 1106 arrays representing three primary cultures, each recorded eight to nine times over a three-week period were examined. From this dataset across all recordings, 75,390 STTC values were calculated from 32,414 unique pairs of neurons. Of the 75,390 STTC values calculated from the data, 43,421 (57.6%) were deemed to represent functional connections based on permutation testing. This selection was made with the qualification that these relationships are based on firing patterns and not detection of physical connection.

**Figure 11.**
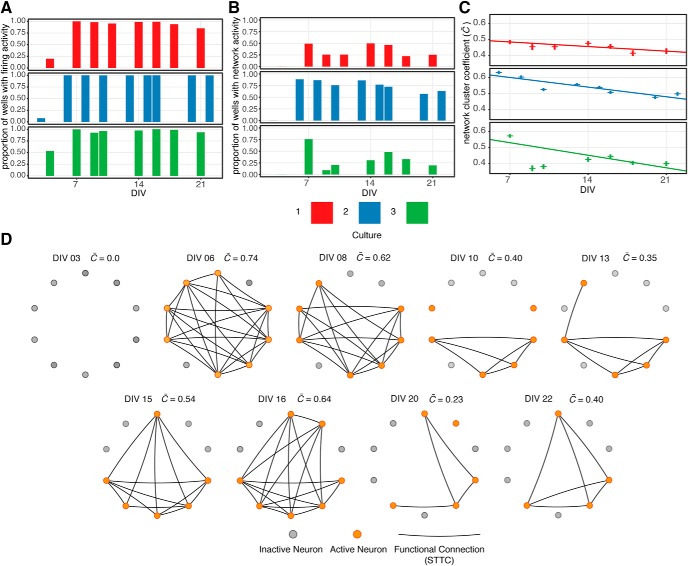
Pattern of network activity within cortical cultures. ***A***, Proportion of arrays across three cultures exhibiting spontaneous firing activity recorded between DIV3 and DIV22. ***B***, Proportion of arrays across three cultures exhibiting network activity as determined by non-zero, array level average network correlation coefficient. ***C***, Change in average network correlation coefficient between DIV3 and DIV22. Bars indicate mean ± SE, regression fit derived from linear mixed model. ***D***, Depiction of network topography between 10 neurons within a single well (array) across nine recordings. Activity state of neuron within recorded indicated by vertex color, lines indicate functional connection between neurons as determined by STTC of significant magnitude.

Specifying these connections, the topography of the functional network between neurons detected on each array during each recording session can established. to quantify the degree of connectedness within each network, the network cluster coefficient ($\bar{C}$) is calculated for each array and recording (Watts and Strogatz, 1998). Examining the activity across these 1106 arrays reveals that spontaneous firing activity does not always result in the detection of network activity. As shown in Figure 11, while the vast majority of arrays exhibit spontaneous firing activity emerging within the first week of culture (panel A), network activity as defined by a non-zero ($\bar{C}$) value is detected in a much lower proportion of arrays. Further, the density of the networks as determined by ($\bar{C}$) significantly decreases from a peak within the first week at an estimated rate of –0.008/d (*p* < 0.001; Fig. 11*C*). This decline in network activity is consistent with findings reported by Golshani and colleagues showing that firing patterns of mouse cortical neurons *in vivo* become less synchronized over a period comparable with the age of these *in vitro* cultures (Golshani et al., 2009). However, given the relatively simple topography of the networks detected in these cultures caution should be taken when drawing conclusions based on changes in density parameters. Further, despite this seemingly steady decline in network density with maturation of cultures, the topography within individual networks can be seen to change dramatically between recordings as shown in Figure 11*D*.

### Paradigms of spike cluster firing patterns

Given the stochasticity observed in the firing patterns of spike clusters over successive recordings, the firing pattern of any one spike cluster can be thought of as adhering to one of four paradigms: (1) persistent, (2) lost, (3) recovering, or (4) emergent. (1) persistent, pertaining to clusters active during all recordings of interest; (2) lost, pertaining to those that are active during initial recordings but not observed in subsequent recordings, either as the result of chance or the consequence of an experimental perturbation; (3) recovering, pertaining to those that are active during initial recordings, inactive during interim recordings, yet are observed again in subsequent recordings, similarly either as the result of chance or the consequence of an experimental perturbation; and lastly, (4) emergent, pertaining to those clusters that are absent during initial recordings but observed during subsequent recordings. The emergent class harbors the caveat that these clusters may have been absent during initial recordings due to chance, or that the cluster represent waveforms from a cell active during an initial recording but then as a result of experimental perturbation produces waveforms with sufficiently different shape characteristics as to result in the identification of a distinct cluster than those produced originally. In this potential scenario, the analysis would identify two distinct clusters, mutually exclusive with respect to time, in which one is lost and the other is emergent. These classifications of firing patterns are not purely semantic, since there are practical implications for which classes can contribute to the analysis of different phenotypes. For frequency-dependent phenotypes, such as rates of firing, the absence of activity is itself relevant. However, for frequency-independent phenotypes, such as wave form amplitude (discussed below), the phenotype is dependent on activity such that the analysis needs to be restricted to those persistent clusters that present during all recordings when these frequency-independent phenotypes are being assessed.

### Examining changes in firing frequency and voltage potential amplitude in response to exogenous treatment

To examine changes in two distinct phenotypes, (1) spontaneous firing frequency and (2) magnitude of voltage potentials, cultures were treated with two well characterized neurotoxins with different mechanisms of action. A pore-forming toxin, *α*HL derived from *Staphylococcus aureus* (Parker and Feil, 2005) and the Na^+^-channel blocker TTX (Narahashi et al, 1964). A pore-forming toxin was chosen for this analysis since an increase in conductance across the membrane resulting from perforation an ionophore such as *α*HL would be expected to cause a decrease in wave form amplitude.

### Effect of neuronal toxin treatment on cell viability

**Figure 12.**
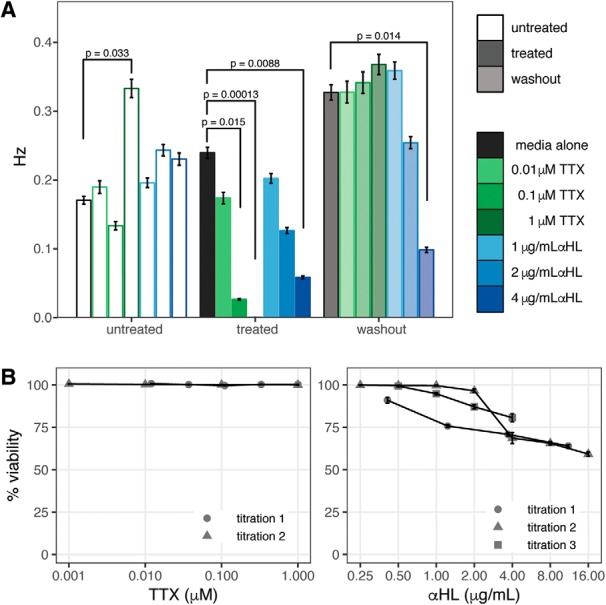
Spike cluster firing frequencies within TTX, *α*HL-treated cultures. ***A***, Firing frequencies (Hz) of spike clusters within cortical cultures treated with titration of TTX (green) or *α*HL (blue). Frequencies from recordings across three times points: untreated (open bars), 24 h post treatment (filled bars), and washout 24 h following removal of treatment (half-filled bars). Treatment of 22–39 replicate wells per condition across four experiments. Data presented as mean ± SE Hz obtained from estimates of Γ-GLM modeling frequency as function of treatment and recording. Differences in frequencies assessed by general-linear hypothesis test, significance indicated by brackets and associated *p* values. ***B***, Percentage viability of cortical cultures following 24-h treatment with TTX (left), or αHL (right) as assessed by LDH release. Data presented as mean ± SE, *n* = 5 per condition.

To determine the maximum tolerated dose of these two toxins, a cell viability assay was performed to assess the induction of cell death in cultures following treatment with a titration of each agent. Cortical cultures were treated with titrations of *α*HL (0.25–16 μg/ml) and TTX (0.001–1 μM) for 24 h, after which viability was assessed by detection of lactate dehydrogenase (LDH) released into the culture media. As shown in Figure 12*B*, *α*HL was tolerated up to 4 μg/ml causing an ∼30% reduction in viability, while TTX had negligible effect on viability even at the highest dose tested. Based on these data, *α*HL was used at a maximum concentration of 4 μg/ml in all subsequent experiments.

### Effect of neuronal toxins on firing frequency

To assess whether these concentrations of TTX and *α*HL were sufficient to cause an effect on the generation of action potentials, the frequency of spontaneous firing was assessed in cultures treated with a titration of TTX or *α*HL at 24 h following treatment, and again at 24 h following the removal of treatment (washout). The data were modeled using a Γ-GLM, estimating the average Hz across the spike clusters within each condition as a function of treatment and recording. Differences in firing frequencies between conditions were assessed using a general linear hypothesis test (Hothorn et al., 2008). As shown in Figure 12, treatment with 0.1 μM TTX (0.0266 ± 0.001 Hz) and 1 μM TTX (0.000556 ± 2e-5 Hz) as well as 4 μg/ml *α*HL (0.05885 ± 0.002 Hz) resulted in a significant decrease in firing frequency compared to treatment with media alone (0.24 ± 0.008 Hz), *p* = 0.0148, *p* = 0.0001, and *p* = 0.0088, respectively. While 2 μg/ml *α*HL (0.127 ± 0.004 Hz) also appeared to induce a decrease in firing relative to control, the difference was not significant, *p* = 0.234. Following the washout of treatment, the TTX-treated conditions show a complete restoration of firing activity, returning to levels comparable with that of media alone, while firing within the 4 μg/ml *α*HL treatment condition was still significantly reduced compared to media alone, *p* = 0.014. While the firing frequency within the 4 μg/ml *α*HL condition increased following the removal of treatment, to 0.098 ± 0.004 Hz from 0.059 ± 0.002 Hz, suggestive of some recovery, this increase was not significant, *p* = 0.82.

The observed decrease in firing frequency among the 4 μg/ml *α*HL-treated condition following treatment could be partially attributable to the effect on cell viability at this concentration (Figure 12*B*), given that the effect was seen both in the presence of the toxin and sustained following its removal. This analysis examined the average firing frequency across all clusters active during each recording, and not specifically the change in firing frequency among cluster that showed persistent activity across recordings. Performing the analysis on the aggregate of all clusters is necessary to capture the effect of a treatment with the magnitude of effect of 1 μM TTX, in which there are no active clusters in the treated condition in which the comparison can be made.

### Effect of neuronal toxin on voltage potential amplitude

Having examined changes in firing frequency at the cluster level, the next question was whether it is possible to assess disruptions in membrane integrity by assessing changes in the amplitude of voltage waveforms emitted from the generation of action potentials.

**Figure 13.**
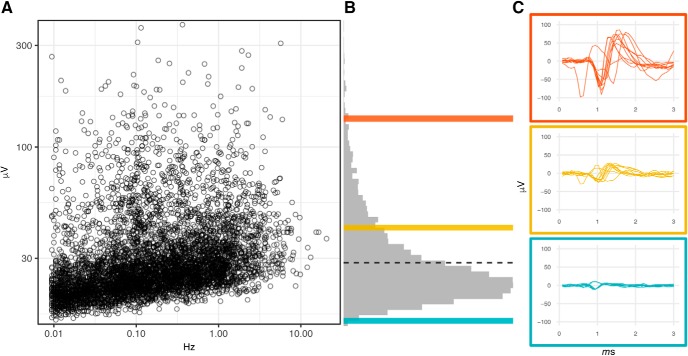
Comparison of firing frequency and wave form amplitude. ***A***, Firing frequency (Hz) and wave form amplitude (μV) for 5506 spike clusters within untreated cortical cultures. Wave form amplitude determined by cluster centroid. ***B***, Distribution of spike cluster wave form amplitudes, dash line indicating 30-μV threshold used for selecting clusters for assessing changes in wave form amplitude in response to treatment. ***C***, Examples of low amplitude (teal), mid amplitude (gold), and high amplitude (persimmon) amplitude waveforms.

There is minimal correlation between the rate at which a neuron fires and the detected amplitude of the resulting voltage potential Figure 13*A*. This lack of correlation stems from the different factors underlying the generation of these properties and the manner in which they are detected in this system. The firing frequency of a neuron, absent any exogenous experimental stimulus, arises from the intrinsic properties of that particular class of neuron and the excitatory and inhibitory inputs it receives from other neurons that have formed synapses on it (Bean, 2007). Conversely, while the action potentials of all neurons are expected to be of the magnitude of ∼70–100 mV with respect to the charge disparity between the intra-cellular and extracellular space, the potential that is detected by the extracellular recording electrode of the MEA is dramatically influenced by the physical constraints of the system. If a firing neuron is modeled as a point source of charge, the extracellular potential *V_e_* could be approximated from Coulomb’s law, such that:

$$V_{e}=\frac{I}{4\pi d\sigma },$$

where I is amplitude of a point source of current (A), and d is the distance from the source (meters), and *σ* is the conductivity (Siemens/meters) of extracellular space (Gold et al., 2007). However, since neurons are better approximate by cylinders whose length is much greater than their transectional radius, the model is refined by the linear source approximation (LSA) described by Holt and Koch (1999). Thereby approximation of the extracellular potential is instead modeled as:

$$V_{e}=\frac{I}{4\pi \sigma \Delta s}log\left|\frac{\sqrt{h^{2}+r^{2}}-h}{\sqrt{l^{2}-r^{2}}-l}\right|,$$

where Δ *s* is the length of the cylinder, *r* is the radial distance from the cylinder, *h* is the longitudinal distance from the end of the cylinder, and *l* = *h* + Δ *s* representing the longitudinal distance from the beginning of the cylinder (Holt and Koch, 1999; Gold et al., 2006, 2007). The practical consequence of this is that the wave form of extracellular voltage potential will vary drastically depending on the spatial orientation with respect to and distance between the firing neuron and the recording electrode, with an amplitude in the range of ∼10–100 μV, two to three orders of magnitude below the potential across the cell membrane (Gibson, 2012; Buzsáki et al., 2012). Taken together, these considerations explain the wide range in firing frequencies (Hz, *x*-axis) and voltage amplitude (μV, *y*-axis) observed across this population of spike clusters.

Since an increase in conductance across the membrane resulting from perforation by *α*HL would be expected to cause a decrease in wave form amplitude, the analysis was limited to spike clusters exhibiting a potential of at least 30 μV before treatment to allow for a sufficient dynamic range of response. This threshold is indicated by the dashed line on the histogram in center of Figure 13. Additionally, in an attempt to estimate the magnitude of the change in amplitude on individual cells, the analysis was limited to those persistent clusters that were active during both the untreated and treated recordings.

**Figure 14.**
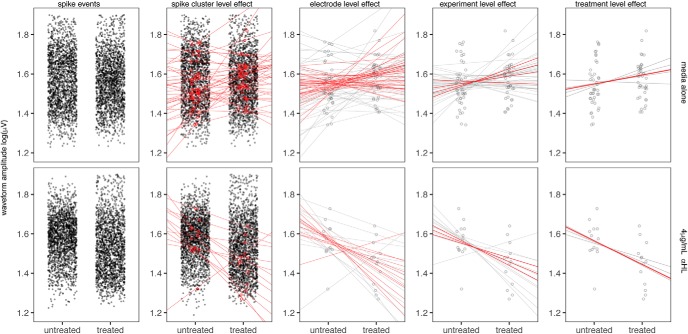
Determining change in wave form amplitude using linear-mixed model. Modeling effect on wave form amplitude in response to treatment media alone (top row) or 4 μg/ml αHL (bottom row) across untreated and treated recordings. Estimates derived from amplitude values of individual waveforms (sample of 2000 shown per condition), and refined at level of spike cluster (open circles), electrodes, experiment, and treatment, indicated above each panel. Estimates at each level shown in red, with estimates of subsidiary level shown in gray.

The estimate of the effect of treatment on wave form amplitude has to account for several factors within the data. First, the estimate of the amplitude of a given neuron during a recording period is derived from the random sample of firing events detected from that neuron, which will exhibit a certain degree of variability. Next, the amplitude across spike clusters varies greatly both before the initiation of treatment, and likely in response to treatment. Additionally, multiple spike clusters can be detected by a single electrode. Each electrode will exhibit a range of sensitivity due to either the micro-environment within the culture in which they reside, and/or in the physical circuitry connecting them to the recording device. Finally, these data were collected across several experiments relying on separate preparations of cultures and reagents, which can contribute to additional variability. Therefore, to obtain an estimate for the effect of each treatment on wave form amplitude while accounting for these sources of variability, a linear mixed-effect model (LME) was constructed to predict *log*_10_μV as a function of the fixed effects of treatment and recording (untreated and treated), while controlling for cluster, electrode, and experiment as nested-random effects (Pinheiro and Bates, 2000). The construction of the model is demonstrated in Figure 14 for two treatments, media alone and 4 μg/ml *α*HL. Based on the raw data of individual waveforms, a series of linear regressions are fit, first based on waveforms within an individual spike cluster, then for all clusters detected by a common electrode, all electrodes within a single experiment, and finally for the effect of treatment across all experiments.

**Figure 15.**
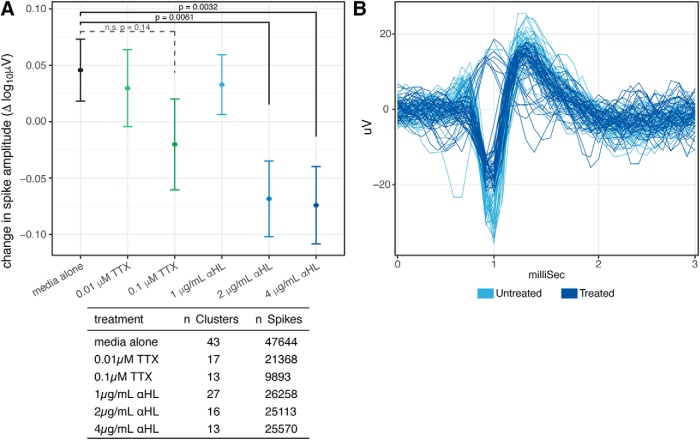
Change in wave form amplitude following treatment with pore-forming toxin. ***A***, Estimated of change in spike wave form amplitude (Δ*log*_10_ μV) of spike clusters within cortical cultures treated with titration of TTX (green) or αHL (blue), across untreated (0 h) and treated (24 h) recordings. Treatment of 22–39 replicate wells per condition across four experiments. Data presented as mean ± SE Hz obtained from LME model of wave form amplitude as function of treatment and recording. Differences in wave form amplitude assessed by general-linear hypothesis test, significance indicated by brackets and associated *p* values. Table indicates number of spike clusters, and spike events available for analysis in each condition. ***B***, Comparison of waveforms within spike cluster demonstrating the largest change in amplitude (μV) among the 4 μg/ml *α*HL treatment condition. Sample of 50 waveforms per recording shown, untreated (light blue) and treated (dark blue).

This method was applied to the recording data from treatment of cultures with a titration of TTX and *α*HL, to determine if an increase in membrane conductance resulting from exposure to a pore-forming toxin would result in a decrease in the amplitude of the voltage waveforms detected. The results in Figure 15 show that treatment with either 2 μg/ml *α*HL (–0.068 ± 0.034 Δ *log*_10_μV) or 4 μg/ml *α*HL (–0.074 ± 0.034 Δ *log*_10_μV) induced a significant decrease in wave form amplitude compared to treatment with media alone, *p* = 0.006 and *p* = 0.003, respectively. While the media alone condition appeared have a slight increase in wave form amplitude (0.046 ± 0.027 Δ*log*_10_μV), this estimate is not significantly different from zero, *p* = 0.09. While treatment with 0.1 μM TTX (–0.02 ± 0.04 Δ*log*_10_μV) showed a slight decrease in wave form amplitude as well, this difference was not significant, *p* = 0.1. Treatment with 1 μM TTX could not be included in this analysis, since this concentration caused complete inhibition of firing activity.

It is worth noting that the significant effects on wave form amplitude are somewhat modest. The estimated change of –0.074 Δ*log*μV within the 4 μg/ml *α*HL-treated cultures translates to a potential difference on average of –5.74 μV after accounting for the baseline amplitude among the spike clusters. That said, due to the attenuation of voltage potentials detected by extracellular electrodes as a result of distance from the neuron and resistance of the extracellular milieu the decrease in potential across the cell membrane is probably a 100× greater or more (Buzsáki et al., 2012). Despite this attenuation of signal, the resulting decrease in amplitude is still apparent. Figure 15*B* shows waveforms from the spike cluster within the 4 μg/ml *α*HL treatment condition exhibiting the largest change in amplitude (Δ 14.2 μV) between the pre-treatment and post-treatment recordings.

Lastly, is important to emphasize that despite treatment with 4 μg/ml *α*HL inducing a slight decrease in cell viability (Figure 12*B*), any such decrease in viability would not be consequential for the effect on wave form amplitude, since these estimates were derived purely from those clusters active during both the untreated and treated recordings.

## Discussion

Multi-well, MEAs are a compelling assay platform for neuroscience combining a label-free, functional, electrophysiological read-out with the ability to multiplex experimental conditions. The increased commercial availability of these instruments over the last decade has led to wider adoption and an increase in published reports of MEA-based experiments. However, despite this increased use, there are limited studies delineating rigorous assay development and experimental methods for use of these instruments.

Having generated recording data from a large population of over a thousand MEAs, firing frequencies at the array level were examined, revealing a highly skewed and highly variable distribution. It was shown that application of log-transformation should be used to obtain approximately normal distributions of firing frequencies before statistical analysis, and that implementation of a bootstrapping simulation can be used to accommodate for the broad distribution of firing frequencies to generate treatment with comparable levels of activity. Next, a series of simulated experiments were performed to determine the expected statistical power for a two condition, repeated measure experiment across a range of sample and effect sizes, based on the observed distribution of firing frequencies of arrays and the empirically determined correlation of firing in repeated recordings.

Assessing several methodological aspects of MEA experiments, many of the commonly reported methods appear adequate for estimating firing activity within *in vitro* neuronal cultures, while other elements of MEA experimental design and analysis have either been under-reported in the literature or investigators have employed techniques that may be inappropriate for the task. Specifically, recording duration of 20 min or longer as reported by several studies (Kuperstein et al., 2010; McConnell et al., 2012; Vincent et al., 2013; Slomowitz et al., 2015; Black et al., 2018; Feng et al., 2018), appear sufficiently long to capture the activity in individual arrays with a high degree of reproducibility. Similarly, the common practice of performing experiments with primary cultures that have been aged two to three weeks is reasonable, since the primary rat cortical cultures examined here showed a high degree of spontaneous firing by the end of the first week that plateaued through these time frames. Conversely, the highly skewed distribution of firing frequencies across both individual electrodes and entire arrays has been largely omitted from reports or accounted for in presentation of results, with a limited number of noted exceptions (Biffi et al., 2013; Vincent et al., 2013; Slomowitz et al., 2015; Wainger et al., 2015; Black et al., 2018). Despite this observation, several of the reviewed publications used parametric statistical tests such as Student’s *t* test and ANOVA for assessing differences in firing frequency between MEAs. This is concerning given that these tests assume a normally distributed dependent variable, a condition that these data fail to meet when examined on a linear scale. Further, some studies used these methods for analyzing longitudinal experiments (Feng et al., 2018; Sarkar et al., 2018), which similarly violate the underlying assumptions of the common forms of these tests which expect measurements to be independent and do not account for the anticipated correlation of values between repeated measurements of the same subject. Rather, ANCOVA and other linear regression based methods that handle time as covariate within the data would be more appropriate (Diggle et al., 2002; Singer and Willett, 2003), as was employed here for the simulation of treatment or as was reported by Wainger et al. (2015).

Despite the increasing number of studies using multi-well MEAs, none of the reports examined described methods for accounting for the wide variability of firing frequencies when assigning treatment groups. Given the ability to easily record MEAs repeatedly, implementation of a simulation-based assignment technique using baseline recording data such as described here seems adequate for the purpose. While the use of complex treatment maps generated by the process may seem impractical for the addition of experimental perturbations, it is certainly feasible for experiments being performed manually, and is trivial for MEA recording systems that are integrated with automated liquid-handlers as is the case with instruments such as the Maestro Apex (Axion Biosystems, Hamilton Robotics).

Using simulation methods incorporating data on the variability and reproducibility of MEA recordings to calculate statistical power of experiments showed the magnitude of effects that can be detected with confidence this system. Comparing these simulated results with empirical data from treatment of cultures with the neurotoxin TTX shows that it is possible to detect changes in firing frequency similar to those induced by treatment with TTX at its IC_50_ using treatment groups containing eight to 10 replicates. Conversely, detecting large changes in firing, such as those induced by high doses of TTX (10× IC_50_), only requires a few replicates, while detecting more modest changes will require many more replicates. This is an important consideration for experimental design in the different research areas in which MEAs are being used. For toxicology studies in which the effect sizes of pharmacological agents maybe larger, a few replicates per condition may suffice, compared to disease modeling studies assessing the functional effects of genetic variants where the effect sizes are expected to be smaller. For these studies, much larger numbers of arrays would be required to discern statistically significant differences.

Further, the high-frequency (>10 kHz) sampling rates of typical MEA instruments can allow for greater resolution of recording data, attributing events to individual cells of origin through the use of spike sorting analysis. Doing so can allow for the data acquired through MEA recordings to be extended beyond aggregate array-level (well-level) activity metrics to more refined phenotypes including changes in: the spiking frequency of individual cells, the amplitude of action potentials, and the network activity neurons revealed through coordinated firing patterns. Through this work, a spike sorting analysis pipeline was developed implemented in the open source R statistical programming language which provides flexibility to perform spike sorting across data from multiple recordings, and that it can be executed within a parallel computing environment, using multiple CPUs within a high-performance computing cluster. The ability to perform spike sorting across multiple recordings is an important distinction of the pipeline developed here relative to other commercially available software. This is important, given the longitudinal nature of many MEA experiments, and the problems with sorting separately for each time point. Further, the ability to leverage parallel computing is advantageous given the large number of recording channels to be assessed (768 within the instrument used for this study) and the time and computationally intensive nature of the mean-shift algorithm underlying the analysis.

Utilizing this analysis pipeline, untreated cultures examined over multiple days were revealed to exhibit highly stochastic patterns of spontaneous firing among individual cells. This observation provided essential grounding for how to draw inferences about changes in firing patterns across populations of spike clusters, as demonstrated with the use of Γ-GLMs. Additionally, to assess changes in the amplitude of action potentials originating from individual cells, spike sorting analysis was performed on recording data of cultures treated with a pore-forming toxin, *α*HL, and non-pore forming, channel blocking toxin, TTX. This analysis showed an significant decrease in the voltage potentials within spike clusters of cultures following treatment with *α*HL, consistent with the expected changes in voltage potentials resulting from perforation of the cellular membrane. This type of analysis may have broader application in the field of toxicology where MEA systems are commonly used. The technique described here for monitoring changes in action potential amplitude is analogous to the methods used for detecting changes in QT intervals in cardiomyocytes recorded using MEAs (Tertoolen et al., 2018). Finally, using these data in conjunction with the STTC method described by Cutts and Eglen (2014) and network cluster coefficient described by Watts and Strogatz (1998), it was shown how the network activity within cultures can be monitored using MEA recordings by assessing the changes in coordinated firing across individual neurons. While previous studies have examined network dynamics within cultures on *in vitro* MEAs, these have typically been carried without first identifying individual cells through spike sorting and instead have examined the temporal correlation of events at the electrode level (Vincent et al., 2013). This obfuscates elements of the network, since activity of multiple cells is likely being aggregated on a single electrode. Incorporating the techniques describe here will facilitate experiments assaying for changes in network dynamics rather than simply aggregated spike frequency. The ability to use network phenotypes as an assay readout will improve as multi-well MEA systems with higher electrode density become available allowing for better resolution of network activity *in vitro*. This is relevant for modeling disease biology, as the changes in neuronal activity resulting from the pathophysiology of conditions such as Alzheimer’s disease are likely to manifest as aberrant firing patterns rather than simply an increase or decrease in the frequency of spiking events (Palop and Mucke, 2010).

## Synthesis

Reviewing Editor: Jorge J. Palop, Gladstone Institutes and UCSF Decisions are customarily a result of the Reviewing Editor and the peer reviewers coming together and discussing their recommendations until a consensus is reached. When revisions are invited, a fact-based synthesis statement explaining their decision and outlining what is needed to prepare a revision will be listed below. The following reviewer(s) agreed to reveal their identity: Kenneth Kosik.

SYNTHESIS

In this manuscript, the authors review the analysis of MEA data performed in previously published works and discuss the best ways to analyze MEA recordings. The authors discuss several important limitations of the MEA approach and they offer information and guidelines on ways to address these limitations. This information will be important to share with the broader neuroscience community and with scientists who are interested in beginning to work with MEA's in particular. However, the reviewers also identified several concerns that need to be addressed.

1) Neuronal density and array density seem to be two key variables that should greatly influence the quality and metrics of collected MEA data and are not experimentally addressed. Do high array density or optimal culture density address some of the experimental caveats discussed (e.g., data distribution, inactive wells, etc.)? It seems important to address this experimentally.

2) The authors raise concerns about the statistical analyses of median firing rate (MFR) data, but they also should indicate which statistical analyses should be used for MFR (parametric vs non-parametric), and which analysis should be used for the log 10 transformed data.

3) Considering the finding that there are many so inactive clusters (up to 40%) in each culture (Figure 8), are the array-level recordings meaningful for the analysis of treatment groups? Is this problem resolve with high array density or optical culture density?

4) This work suggests that many replicates may be needed to see a significant effect of a treatment. As a comparison, how many replicates have other studies done?

5) The authors identify functional connections based on STTC values as “observations of sufficient magnitude with a <1% probability of occurring by chance”. What is the rationale for using this approach? What does sufficient magnitude mean?

6) The authors recommend criteria for excluding inactive wells and for assigning wells to treatment groups. Have others used similar criteria for excluding inactive wells?

7) They should also support their claim that MEAs are useful to model “Alzheimer's, Parkinson's, schizophrenia, and many other diseases of the human central nervous system” with references.

8) Their extensive presentation on assigning treatment and control groups should be introduced by examples in which MEAs have distinguished two such groups.

9) MEA equipment is now available with very high densities of electrodes, which makes the work presented here seem dated.

10) Their sampling frequency at 12 kHz is a bit slower than some other reports. They should say why they chose this low sampling frequency.

11) Methods are available to detect sub-threshold spikes and because they contribute significantly this should be mentioned.

12) They said: “A pool of active arrays from a multi-well MEAs was established by selecting those arrays that are no more than 2 SD below the median of the sample set.” 2 SD by what parameter? Firing rate?

13) They did 30 min recordings. They need to show how the same culture changed by comparing the first 5 minutes with the last 5 minutes. While this topic is addressed theoretically and by way of review in the section entitled “Duration of recording for achieving intra-array reproducibility” more detail and explicit numbers are needed. Also needed is some sense of intra-array reproducibility as a function of culture age. The brief paragraph on maturation seems to minimize this variable which is inconsistent with other data in the field.

14) They need to compare their spike sorting method with widely used methods. They assert that, “The most suitable product available at the time of initiation of this study was Offline Sorter (Plexon, Dallas TX)” but give no basis for this assertion. The shortcoming described in “Spike sorting in a recording-dependent versus recording-independent fashion” is a serious flaw when comparing data on different using these methods and needs more emphasis. They address the problem by applying the Gamma distribution: “The combination of a large proportion of inactive clusters and a limited number of highly active clusters results in a highly skewed distribution of firing frequencies as a shown in the left panel of Figure 8C, analogous to the distribution firing frequencies observed at the array-level Figure 1.” A deeper explanation is needed for how a Gamm distribution can address this issue or whether it can address the issue and how they validate their conclusion.

15) Whether functional connections between neurons can be established based on the spike time tiling coefficient (STTC) calculated between the spike trains of events attributed to each individual unit needs much more substantiation. How much of the reported in-phase activity, which they say predominates is due bursting. When in-phase activity is noted they need to address the latency of the phase displacement.

16) They compute a correlation coefficient of firing frequencies among a population MEAs recorded at separate times. Were there differences in the burstiness? How do they account for that? We need to see variation within a single MEA over time which might be partially addressed by determining ISIs. It's really not even clear that firing rate is the most informative parameter especially when most of the units are hidden in these low density MEAs.

17) Spike sorting is not the optimal method for identifying individual neurons because a substantial number of the spikes are propagation signals and therefore derive from a single neuron but have diverse wave form morphologies as the axon traverses the array. Please include this caveat.

18) They say: “Of the 75,390 STTC values calculated from the data, 43,421 (57.6 %) were determined to represent functional connections based on permutation testing.” What is the ground truth?

19) They say there is “...steady decline in network density with maturation of cultures...” This sounds counter-intuitive and needs substantiation.

20) The small number of electrodes and low density of the array means the majority of the data is missing. Therefore, it seems misleading to use terms like lost or emergent or recovering spikes.

21) The data on “Effect of neuronal toxin on voltage potential amplitude” seems particularly problematic given the variation discussed above and the variable that wave forms are mainly a function of distance from the electrode. It is not clear that they have the resolution to use waveforms in the manner described.

22) This sentence, particularly the word “extent” needs clarification: “Further, implementation of unsupervised spike sorting to resolve activity to the level individual neurons (single-units) demonstrates how MEA recording data may be extent for examination of waveform amplitude and functional network phenotypes.”

23) This paper might fare better by more completely compiling the literature and sticking to a review format rather than presenting problematic analyses with MEAs that have limited detection utility. This problem might be addressed if they include an example of how their methods distinguish some of the disease conditions mentioned in the abstract.

24) Does the power increased by analyzing before and after treatment instead to between treatments? There is no mention on longitudinal analyzes before and after treatments which may account for some inter-array variability. Page 17 does not include this discussion or data analyses.

25) Raw traces from arrays indicating the threshold for spike detection and exclusion would be useful.

26) Page 13, change “on the order of 10s of minutes” to “10s to minutes”?

27) Include the length of the recordings in the legend of Figure 1.

28) Page 14, text states that recordings are 3 hours, but Figure 2 shows only the results of the correlations up to 2 hours. Please match text and figure.

29) Firing frequency in figure 13 has not been log10 transformed. Earlier, it was concluded that log10 scale is the best approach. An explanation note would be useful.

30) In Figure 3, it is difficult to see the red and blue data points in the graph. The distribution of frequencies recorded in culture 4 (purple data points) seem broader than the distribution of frequencies recorded in culture 3 (green data points). Is this true? Does this add to the variability in firing across wells?

31) Figure 13, the authors seem to be using raw firing frequencies without log transformation despite their concerns raised about raw firing rates. Due to the great variability in the untreated groups, assessing effects before and after treatments would be important to assess,

32) There are many figures can be consolidated to reduce the total number of figures. For example, combine Figures 12 and 14, and Figures 16 and 17. Figure 11 can be deleted since the text is clear by itself.

## Author Response

We thank the reviewers for their thorough review. We have considered all of the comments, and in response have performed additional analyses, and have edited the text of the document to address each of the critiques. Below we respond in a point-by-point manner to each comments, our responses are in bold.

Synthesis Statement for Author (Required): SYNTHESIS

In this manuscript, the authors review the analysis of MEA data performed in previously published works and discuss the best ways to analyze MEA recordings. The authors discuss several important limitations of the MEA approach and they offer information and guidelines on ways to address these limitations. This information will be important to share with the broader neuroscience community and with scientists who

are interested in beginning to work with MEA's in particular. However, the reviewers also identified several concerns that need to be addressed.

1) Neuronal density and array density seem to be two key variables that should greatly influence the quality and metrics of collected MEA data and are not experimentally addressed. Do high array density or optimal culture density address some of the experimental caveats discussed (e.g., data distribution, inactive wells, etc.)? It seems important to address this experimentally.

Early assay development data (not shown) indicated that above a certain plating density, activity was not further increased (perhaps due to additional cells not adhering beyond covering the surface area of the array). We used a plating density (240k/cm^2^) that results in nearly all of the surface area being covered (see Supplementary Content for Reviewers, Fig.1). The range of culture densities reported in the literature for MEA experiments varies broadly, greater than two orders of magnitudes:

Black et al 2018 - 16K/cm^2^; Varghese et al 2010: 20K/cm^2^; Charkhkar et al 2015: 35K/cm^2^; Kuperstein et al 2010: 100K/cm^2^; Wainger et al 2014: 135K/cm^2^; this study: 240k/cm^2^; Feng et al 2018: 312K/cm^2^. However, for many studies the plating density is not clearly stipulated in the methods.

Images of clear-bottom MEA plated at the 240k/cm^2 density show highly confluent cultures, indicating that variability in signal is unlikely to be the result of sparse cultures (see “Supplementary Content forReviewers” Fig. 1). Yet, even within these dense cultures the distribution of neuronal cell bodies is heterogeneous which likely does contribute to the variability in signal observed.

With regards to array density, the density of the array is a fixed parameter of the instrumentation and not possible to manipulate experimentally. A more detailed response regarding array density is provided in response to comment #9.

2) The authors raise concerns about the statistical analyses of median firing rate (MFR) data, but they also should indicate which statistical analyses should be used for MFR (parametric vs non-parametric), and which analysis should be used for the log 10 transformed data.

The text has been updated to indicate that parametric methods are appropriate for analyzing MEA

activity by mean firing rate following log transformation (see page 13 of revised text).

3) Considering the finding that there are many so inactive clusters (up to 40%) in each culture (Figure 8), are the array-level recordings meaningful for the analysis of treatment groups? Is this problem resolve with high array density or optical culture density?

While substantial proportions of individual clusters (neurons) are inactive during specific recordings (Figure 8), the high correlation of array level activity as a function of recording length (Figure 2) suggests that the aggregate activity of the array is consistent if observed over a sufficient period of time (30 minutes or more). That said, higher density arrays are likely to be less sensitive to fluctuations in array level activity resulting from the stochastic firing patterns of individual clusters by virtue of being able to sample from a larger number of cells at time. However, the expectation is that if this analysis were repeated on an array with a higher electrode density, the proportion of clusters inactive at a given time would be similar to that observed here.

4) This work suggests that many replicates may be needed to see a significant effect of a treatment. As a comparison, how many replicates have other studies done?

Many MEA studies unfortunately do not report the number of replicates in a transparent manner, and others actually count different electrodes in the same well as replicates, even though the data from these is interdependent. More rigorous studies have reported effects of known bioactive compounds or disease associated mutations using upwards of 8-10 replicates per condition in MEA based experiments. The text has been updated to indicate these prior reports (see page 18 of revised text).

5) The authors identify functional connections based on STTC values as “observations of sufficient magnitude with a <1% probability of occurring by chance”. What is the rationale for using this approach? What does sufficient magnitude mean?

The text has been edited to clarify the intent of the statement, namely, that following permutation testing, only those observed STTC values that were of sufficiently strong correlation (positive or negative) that the likelihood of occurring by chance is <1% were taken to represent functions connection between neurons (see page 24 of revised text).

6) The authors recommend criteria for excluding inactive wells and for assigning wells to treatment groups. Have others used similar criteria for excluding inactive wells?

It is not clear what other studies have done with regards to excluding inactive wells or assigning treatment wells. The studies we have found either do not do something similar or else do not report this information. The lack of transparency in reporting on some MEA studies is one stimulus for providing a report such as this that describes best practices. Some studies including that by Bardy et al 2015 have stated criteria for considering an individual electrode to be active, a firing rate >0.005Hz in that case, but this was not extended to the well level.

7) They should also support their claim that MEAs are useful to model “Alzheimer's, Parkinson's, schizophrenia, and many other diseases of the human central nervous system” with references.

The text of the abstract states “are being more widely used by researchers ... for applications in modeling Alzheimer's, Parkinson's, schizophrenia ...”. This is merely a statement that others have used these instruments for this purpose, which is supported by references made throughout the paper. This is not a value statement claiming the “usefulness” of these instruments for modeling these conditions. Specific references were omitted in the abstract of the manuscript to comply with the required word count.

8) Their extensive presentation on assigning treatment and control groups should be introduced by examples in which MEAs have distinguished two such groups.

It is unclear what the reviewer is asking for in this statement. If clarification can be provided we will happily try to address the concern.

9) MEA equipment is now available with very high densities of electrodes, which makes the work presented here seem dated.

This work is focused exclusively on the use of multi-well in vitro MEA systems. The specifications of the current commercially available multi-well MEA systems are fairly similar to that used herein, detailed in the table below. The one exception to this is the Maxwell Biosystems MaxTwo systems which employs CMOS chip technology and supports 1024 recording channels per well up to 24 wells. However, the multi-well Maxwell instrument was only just commercially released recently (Spring 2019). The considerations for experimental design for MEA discussed in this paper will be relevant for those using these newly available instruments as well, the text of the manuscripthas been revised to emphasize this point (see page 34 of revised text).

While higher density in vitro MEAs such as the 4096 channel, 42µm pitch arrays from ALA Scientific or 3Brain are available, these are single chamber systems, thereby making it difficult to scale to experimental designs that require multiple independent replicates (the focus of this study). Similarly, in vivo shank style MEA have been reported with pitch densities down to 11µm (see Scholvin, J. et al., 2016. IEEE Transactions on Biomedical Engineering, 63(1), pp.120-130.) However these are custom fabricated, non-commercially available systems, and not the type on instrumentation that this study is focused on.

The data presented here show how MEA systems such as the Axion system - which are in broad usage - can be analyzed in a manner that provides meaningful data for studying activity in vitro. Further, the lessons learned here also are applicable to higher density arrays that are only very recently commercially available.

10) Their sampling frequency at 12 kHz is a bit slower than some other reports. They should say why they chose this low sampling frequency.

This sampling frequency was used as it is the maximum sampling frequency of the Axion Maestro instrument used in the study. The text of the methods section has been updated to reflect this fact (see page 6 of revised text).

11) Methods are available to detect sub-threshold spikes and because they contribute significantly this should be mentioned.

The reviewer is correct that sub-threshold potentials can be recorded using MEAs. The text has been revised to include this point (see page 12 of revised text).

12) They said: “A pool of active arrays from a multi-well MEAs was established by selecting those arrays that are no more than 2 SD below the median of the sample set.” 2 SD by what parameter? Firing rate?

Yes, only those arrays which exhibited a firing frequency no more than 2 SD below the median firing frequency of the sample set were included for analysis. The text has been updated to clarify this point (see page 7 of revised text).

13) They did 30 min recordings. They need to show how the same culture changed by comparing the first

5 minutes with the last 5 minutes. While this topic is addressed theoretically and by way of review in the section entitled “Duration of recording for achieving intra-array reproducibility” more detail and explicit numbers are needed. Also needed is some sense of intra-array reproducibility as a function of culture age. The brief paragraph on maturation seems to minimize this variable which is inconsistent with other data in the field.

The reviewer seems to be referring to the results shown in Figure 2 demonstrating the correlation of firing frequencies across multi-well MEAs recorded on consecutive days. The purpose of this analysis was to demonstrate that the 30 minute recording intervals appears sufficient to account for day-to-day variability in firing frequency when performing multi-day experiments. This recording duration is in line with the methods reported by other studies as detailed in Table 2.

To address the reviewers comment we have included additional analysis comparing the correlation between the first 5 minutes and final 5 minutes as well as the first 30 minutes and final

30 minutes of 3hr recordings performed on consecutive days (see supplementary content for reviewers Fig. 2). As would be expected, the correlation is higher when comparing intervals within the same day recording than when compared across days. Further, the correlations are higher when comparing 30 minute intervals than when examining only 5 minutes. Further we have compared the correlation in firing activity across 30 minute recording in cultures monitored over several weeks, showing that the activity between recordings becomes more positively correlated with culture maturation (see supplementary content for reviewers Fig. 3).

14) They need to compare their spike sorting method with widely used methods. They assert that, “The most suitable product available at the time of initiation of this study was Offline Sorter (Plexon, Dallas TX)” but give no basis for this assertion. The shortcoming described in “Spike sorting in a recording- dependent versus recording-independent fashion” is a serious flaw when comparing data on different using these methods and needs more emphasis. They address the problem by applying the Gamma distribution: “The combination of a large proportion of inactive clusters and a limited number of highly active clusters results in a highly skewed distribution of firing frequencies as a shown in the left panel of Figure 8C, analogous to the distribution firing frequencies observed at the array-level Figure 1.” A deeper explanation is needed for how a Gamm distribution can address this issue or whether it can address the issue and how they validate their conclusion.

The reviewer's comment refers to several different points made in the manuscript and it is unclear whether comment #14 indicates a misunderstanding of the relationship between these topics, or whether this is a conflation of several comments intended to be handled independently.

The demonstration of recording-dependent verse recording-independent approaches for ascribing events to individual neurons using spike sorting is meant to indicate how performing this

analysis in a recording-dependent manner can result in over segmentation of clusters and

misattribution of events to multiple clusters which in fact are likely arising from a common origin. For this reason, when spike sorting data from multiple recordings, this should be performed in a recording-independent fashion as it was performed in this study. The text has been updated to more strongly emphasize and clarify this point (see page 21 of revised text).

The use of gamma-distributed models for examining differences in cluster-level firing frequencies is completely unrelated to the issue of recording-dependent or recording-independent spike sorting.

Gamma-distributed models were used to examine dynamics of cluster-level firing frequencies across experimental conditions since the empirical data more closely approximates a gamma distribution than other theoretical distributions (Gaussian, Poisson, etc). The appropriateness of approach is determined by examination of the residuals of empirical data with respect to the fitted model. This level of detail is omitted from the text as this is standard practice for all regression techniques and not specific to the topic of the manuscript. Gamma-distributed generalized linear models are simply a commonly used technique for drawing inferences between populations exhibiting this type of distribution, the reference provided (McCullagh and Nelder, 1989) is a standard text describing the use of these models.

The reviewer's point as to why the use of gamma-GLM is recommended for assessing changes in cluster-level firing frequencies and not array-level firing frequencies is well taken. In fact, the use of gamma-GLM is equally appropriate for examining array-level activity as well. Since there is a higher level of basal activity at the array-level (i.e. not zero-inflated) a simple log transformation can be used to approximate a Gaussian distribution and thus allowi one to proceed with more commonly used forms of statistical analysis. While the original text described why the log transformation approach should not be used for the cluster-level data, the revised text does clarify this point, namely that gamma-GLM could be used to assess non-transformed array-level frequency data.

15) Whether functional connections between neurons can be established based on the spike time tiling coefficient (STTC) calculated between the spike trains of events attributed to each individual unit needs much more substantiation. How much of the reported in-phase activity, which they say predominates is due bursting. When in-phase activity is noted they need to address the latency of the phase displacement.

The question about whether functional connections can be established by the STTC is highly relevant. Unfortunately, we cannot definitively determine functional connections based on this measure, but rather differences in the likelihood of connectivity across conditions, using the assumption that nodes with spike times that are substantially overlapping may be connected. Whereas the STTC measure can be strongly affected when firing frequencies are very high (as in sustained burst firing), we examined STTC in the context of bursting to examine whether this is true for our data. To do this, a burst fraction was calculated for each spike train (Mizuseki et al. 2012) and compared the mean burst fraction for the pair of clusters with STTC value associated with each pair (see supplementary content for reviewers Fig. 4). While STTC does increase significantly with increasing average burst fraction (p<0.01), the average burst fraction only explains a small percentage of the variance observed in STTC (11.6%), suggesting the STTC is largely independent of bursting. Finally, the question regarding latency is also very important - to address this, we have plotted spike latencies of spike trains associated with high STTC scores (>0.50) and with low STTC scores (<0.054), to show that the phase displacement is

widely distributed in both cases, suggesting that the STTC measure again is not driven by burstiness (see supplementary content for reviewers Fig. 5).

16) They compute a correlation coefficient of firing frequencies among a population MEAs recorded at separate times. Were there differences in the burstiness? How do they account for that? We need to see variation within a single MEA over time which might be partially addressed by determining ISIs. It's really not even clear that firing rate is the most informative parameter especially when most of the units are hidden in these low density MEAs.

We agree with the reviewer that firing frequencies/rates by themselves are confounded by burstiness, detection limits of the MEA on a given set of plates, and other issues. This is why the ultimate analysis relies on a spike timing measure (STTC), as opposed to a simple correlation of firing frequencies. Although we found the firing frequency correlations to be informative in showing the overall variation of the firing data, we have downplayed its relevance to the analysis in the text. Finally, there are differences in burstiness among different nodes, and we assessed the influence of burstiness in the skewing/biasing the STTC measure. As discussed in response to the comment above, by demonstrating that STTC is largely independent of burst index we show that the finding is still robust.

17) Spike sorting is not the optimal method for identifying individual neurons because a substantial number of the spikes are propagation signals and therefore derive from a single neuron but have diverse wave form morphologies as the axon traverses the array. Please include this caveat.

We thank the reviewers for this comment, and have included this caveat in the text (see page 18 in revised text).

18) They say: “Of the 75,390 STTC values calculated from the data, 43,421 (57.6 %) were determined to represent functional connections based on permutation testing.” What is the ground truth?

As stated above in response to comment #15, we cannot definitively determine functional connections based STTC, but rather the assumption is made that nodes with spike times that are substantially overlapping may be connected. The text has been updated with a caveat to stress this qualification to the reader (see page 24 in revised text).

19) They say there is “...steady decline in network density with maturation of cultures...” This sounds counter-intuitive and needs substantiation.

The text has been revised to emphasize that given the relative simplicity of these networks drawing conclusions from the parameters of these networks needs to be done with caution. However, while declining network density with culture maturity may be seen as counter intuitive, this result is consistent with findings that firing patterns of mouse cortical neurons in vivo become less synchronized over the same period as these cultures (Golshani et al 2009). We have added this information to the text. The purpose of this analysis is to demonstrate a network level parameter that can be calculated from these data and used as an assay readout.

20) The small number of electrodes and low density of the array means the majority of the data is missing. Therefore, it seems misleading to use terms like lost or emergent or recovering spikes.

This reviewers critique regarding array density was previously addressed in response to comment

#9. The density of the arrays notwithstanding, we respectfully disagree that the terminology used is missing leading or that the observations are the result of 'missing data'. Given the high density of the cultures, 240K cell/cm^2 and age of the cultures during the experiments in question (>DIV

21), we do not expect positions of individual cells with respect to a given electrode to change

substantially. Therefore, it is more likely that attenuation or emergence of signal from a cluster is the result of spontaneous changes in firing patterns of a stationary neuron rather than migration away from or into the detection area of the electrode. Since the clustering analysis was performed across recordings, it is reasonable to conclude those instances classified as recovering represent the same cell of origin since the waveforms would differ in shape and resulting cluster

designation if the signal arose from separate cells exiting and entering the detection area. We have provided a clarification in the text on this point, but we have included the caveat that there may be some cell movement that contributes to the loss or gain of signal.

21) The data on “Effect of neuronal toxin on voltage potential amplitude” seems particularly problematic given the variation discussed above and the variable that wave forms are mainly a function of distance from the electrode. It is not clear that they have the resolution to use waveforms in the manner described.

The analysis of changes in voltage potential amplitude was limited to those clusters active in recordings both before and after treatment. As stipulated in the response to comment #20, the clustering analysis was performed across recordings and thus the signal from these 'Persistent' clusters is highly likely to represent signal from the same cell of origin. Therefore, a reasonable interpretation of that data is that the effects on waveform amplitude are the result of toxin treatments across recordings. The alternative explanation suggested by the reviewer that these data are the result of movement of the cells with respect to the electrodes seems dubious as this would require biased migration towards or away from electrodes as a result of treatment to result in the observed effect.

22) This sentence, particularly the word “extent” needs clarification: “Further, implementation of unsupervised spike sorting to resolve activity to the level individual neurons (single-units) demonstrates how MEA recording data may be extent for examination of waveform amplitude and functional network phenotypes.”

The text has been edited replacing “extent” with the intended word “extended” (see page 4 in the revised text).

23) This paper might fare better by more completely compiling the literature and sticking to a review format rather than presenting problematic analyses with MEAs that have limited detection utility. This problem might be addressed if they include an example of how their methods distinguish some of the disease conditions mentioned in the abstract.

This manuscript is meant to be a guide for users intending to model neuronal physiology with multi-well MEA. As addressed in response to comment #7, this work is not meant to demonstrate that these instruments are the best, or better than other experimental techniques for modeling these diseases. The study is meant to show the steps that need to be taken to design robust and reproducible experiments for these systems.

Further, we do not agree with the suggestion that the manuscript would be more appropriate as a review. The original data reported here are necessary to support the recommendations for experimental design and analysis (for ex., those data indicating the necessary number of arrays for sufficient power, or the use of gamma-distributed linear models for assessing the distributions of clusters-level firing frequencies have not been previously demonstrated). This lack of prior art thus precludes the inclusions of these points in a review article. Further, with publication, the

code generated for various steps of the analyses will become freely available to the community, and thus will not need to be created “from scratch” in other labs.

24) Does the power increased by analyzing before and after treatment instead to between treatments? There is no mention on longitudinal analyzes before and after treatments which may account for some inter-array variability. Page 17 does not include this discussion or data analyses.

This is a misunderstanding of the experimental design. All of the analyses discussed under the heading “Adding it all up: Power to detect changes in neuronal firing using MEAs” pp. 16-17 are longitudinal analyses.

25) Raw traces from arrays indicating the threshold for spike detection and exclusion would be useful.

A figure depicting representative raw voltage recordings and crossing thresholds has been added

(Figure 1) in the revised manuscript.

26) Page 13, change “on the order of 10s of minutes” to “10s to minutes”?

The text has been updated to clarify the statement, which should read “tens of minutes” (see Page

12 of revised text).

27) Include the length of the recordings in the legend of Figure 1.

The legend for Figure 1 has been updated to include recording time.

28) Page 14, text states that recordings are 3 hours, but Figure 2 shows only the results of the correlations up to 2 hours. Please match text and figure.

The analysis was performed over repeated 3 hour recordings, however the figure panel was cropped to the initial 2 hours in order focus on the period of most pronounced effect. The text of the Figure 2 legend has been updated to indicate this.

29) Firing frequency in figure 13 has not been log10 transformed. Earlier, it was concluded that log10 scale is the best approach. An explanation note would be useful.

The data shown in Figure 13 are estimates of the firing frequency of populations of spike clusters (single units) in response to treatment with neurotoxins. These estimates are drawn from a Gamma-distributed linear model, as indicated in the text and figure legend. The use of this regression method obviates the need to perform the the log transformation as the highly skewed distribution of untransformed firing frequencies are well approximated by a Gamma distribution. This method was used for reasons described earlier in the text (see heading: Stochastic nature of firing patterns of individual spike clusters) because performing log transformation on the spike cluster level data results in a bi-modal rather than a Gaussian distribution. Given that two reviewers refer to this issue, we have clarified in the text why we used the gamma distribution here and did not perform a log transformation.

30) In Figure 3, it is difficult to see the red and blue data points in the graph. The distribution of frequencies recorded in culture 4 (purple data points) seem broader than the distribution of frequencies recorded in culture 3 (green data points). Is this true? Does this add to the variability in firing across wells?

Figure 3 has been redrawn showing the firing activity of each culture in a separate panel in order to make the effects between cultures more easily distinguishable. In response to the reviewers question as to whether the distribution of firing frequencies differs between cultures, further analysis was performed to assess this question (see supplementary content for reviewers Fig. 6). ANOVA indicates that the standard deviation of firing frequencies is significantly affected by

culture preparation and the age of the culture. Comparison of models indicates that the variance is significantly effected by culture age when controlling for the effect of culture preparation (p =

0.013). Examining the effect of culture preparation indicates that culture 3 (green data points)

does show significantly lower variance than that of cultures 1, 2, and 4 (p = <0.001, <0.001, and

0.0014 respectively). However, there is insufficient evidence to conclude interaction between culture preparation and culture age. Put more simply, the rate of change in variance across firing activity with respect to time appears consistent across cultures. The implications these data have for assay development using MEA platforms are that the variance across arrays within a single preparation of primary cortical cultures appear to reach a minimum between DIV 7 - DIV 14. However, substantial variation across array activity persists during this interval. Using cultures at this age may help mitigate some of the array-to-array variability, but other methods described in this paper such as the bootstrapping simulations for experimental group assignment will still be necessary to fully account for the observed variance.

31) Figure 13, the authors seem to be using raw firing frequencies without log transformation despite their concerns raised about raw firing rates. Due to the great variability in the untreated groups, assessing effects before and after treatments would be important to assess,

Please see response to comment #29 regarding fitting of untransformed data. Further, the data in Figure 11 (Figure 13 in original version) represent repeated measures of the same cultures recorded: before, during, and after washout of treatment. These repeated recordings were accounted for in the regression model fit to the data as indicated in the text, see page 9: “Generalized Linear Models for the distribution of spike cluster frequencies were constructed... Firing frequency (Hz) was modeled as the dependent variable, while treatment and recording were used as interacting categorical independent variables.”

32) There are many figures can be consolidated to reduce the total number of figures. For example, combine Figures 12 and 14, and Figures 16 and 17. Figure 11 can be deleted since the text is clear by itself.

Figures 12, 13 and 16, 17 from the original version have been redrawn as single figures, now figures 12 and 15 in the revised text. Figure 11 from original version has been removed
